# The dynamics of rare earth elements in soil–plant systems in southern Brazil: a case study of carbonatite-bearing areas and *Baccharis trimera*

**DOI:** 10.1007/s11356-026-37896-5

**Published:** 2026-06-11

**Authors:** Lucas Mironuk Frescura, Nicole Werle da Silva, Daniel Triboli Vieira, Maria Luiza de Vargas Mallmann, Edinei Koester, Rogerio Vescia Lourega, Marcelo Barcellos da Rosa

**Affiliations:** 1 Chemical and Pharmaceutical Research Laboratory, Universidade Federal de Santa Maria, Santa Maria, Brazil; 2 Post-Graduate Program in Pharmaceutical Sciences, Universidade Federal de Santa Maria, Santa Maria, RS Brazil; 3 Post-Graduate Program in Chemistry, Universidade Federal de Santa Maria, Santa Maria, RS Brazil; 4 Geosciences Institute, Universidade Federal Do Rio Grande Do Sul, Porto Alegre, RS Brazil

**Keywords:** Rare earth elements, Carbonatite, Soil–plant transfer, *Baccharis trimera*, Geochemistry signatures, REE-bearing minerals

## Abstract

Carbonatite outcrops represent significant global sources of rare earth elements (REEs). However, their influence on surrounding soils, vegetation, and REE biogeochemical cycling remains insufficiently understood, particularly in Brazilian carbonatite-bearing systems. Hence, this study investigated the distribution and transfer of REEs in soils and *Baccharis trimera* near two carbonatite bodies in Caçapava do Sul (Rio Grande do Sul State, southernmost Brazil). Eighty-three samples, including soil, roots, and leaves, were analyzed to determine REE concentrations. Soil analysis revealed significant enrichment in REEs, predominantly comprising light REEs. The soil preserved the geochemical signature of the parent carbonatites, including positive Eu anomalies and no significant Ce anomalies. Monazite, fluorapatite, and pyrochlore were identified as the primary REE-bearing phases. Soil samples from areas closer to the outcrops exhibited significantly higher REE concentrations, whereas more distant samples showed lower concentrations, indicating lithological control. Roots accumulated substantially higher REE levels than the leaves, showing a low bioaccumulation factor and restricted translocation factor, acting as a biological barrier. Strong positive correlations among rock, soil, and plant compartments were observed, confirming that carbonatite lithology directly controls REE distribution across the system. These findings demonstrate the potential of soil geochemistry as a proxy for carbonatite extension and highlight the role of *B. trimera* roots as sinks, preserving the lithological signature of REE-rich systems.

**Supplementary Information:**

The online version contains supplementary material available at 10.1007/s11356-026-37896-5.

## Introduction

Rare earth elements (REEs) are a group of 17 chemical elements, which include Sc, Y, and the lanthanides. Elements from La to Eu are classified as light REEs (LREEs), whereas the remaining lanthanides and Y form the heavy REEs (HREEs). These two groups of REEs typically occur in the same deposits, with the exception of Sc, which is not included in either subgroup (Dushyantha et al. 2020). These elements are highly valued for their strategic significance (Talan and Huang 2022) and critical to modern technologies due to their unique electronic, optical, and magnetic properties, enabling advances in energy, digital devices, catalysis, and environmental applications (Chen et al. 2024; Talan and Huang 2022).

Despite their name, REEs are considerably more abundant in the Earth’s crust (Talan and Huang 2022), often occurring in association with specific minerals such as phosphates, silicates, carbonates, oxides, and halides (Balaram 2022). Thus, carbonatite complexes are a global source of REEs (Liu et al. 2024). Carbonatite is a magmatic rock composed of over 50% carbonate minerals, generally formed from mantle-derived melts (Mitchell and Gittins 2022), and commonly contains REE-bearing minerals, including monazite and fluorcarbonates (Song et al. 2023). Carbonatite-associated deposits represent the primary global source of LREEs, accounting for roughly 51.4% of rare earth oxide resources worldwide (Zheng et al. 2023).

The weathering of carbonatite outcrops can release REEs into the surrounding soils. The distribution and fractionation of these elements reflect the primary mineral dissolution and secondary mineral formation (Bueno et al. 2021). In soils, REE behavior is strongly controlled by physicochemical parameters, including pH, organic matter complexation, clay mineral adsorption, and Fe–Mn oxide adsorption, beyond weathering intensity. These properties impact REE adsorption, desorption, co-precipitation, and surface complexation processes, thereby controlling their mobility and redistribution (Liu et al. 2022). Consequently, soils surrounding carbonatite outcrops often preserve the geochemical signature of the bedrock.

Plants have the capacity to absorb and accumulate metals from the surrounding environment in their above- and below-ground tissues (Rabbani et al. 2024). REE mobility to plants is predominantly influenced by the soil properties previously mentioned, and REE uptake commonly occurs through the roots, although it can also occur through leaves (Turra et al. 2019). Moreover, there are species of plants that thrive in metal-rich soils, allowing them to survive in soils with higher REE concentrations and making them bioaccumulators of these elements. Examples of such species that are REE hyperaccumulators include *Cyperus rotundus*, *Carya tomentosa*, *Phytolacca americana*, *Cannabis sativa*, and *Fagopyrum esculentum* (Rabbani et al. 2024). Despite the growing interest in the interactions between REEs and plants for their effective utilization, research integrating the analysis of soil and plant systems in carbonatite-bearing environments are scarce, particularly in subtropical regions (Cao et al. 2022; Le Jean et al. 2023; Wiche and Pourret 2023; Jin et al. 2025).

*Baccharis trimera* (Less.) DC, popularly known as *carqueja*, is a perennial native species widely distributed in southernmost Brazil. It is abundant in central Rio Grande do Sul State and has an extensive root system, making it a promising species for investigating REE transfer in natural soil–plant systems (Silveira Rabelo and Caldeira Costa 2018; Rabelo et al. 2020). Traditionally, it has been utilized as a medicinal herb to treat renal, intestinal, and gastric disorders, as well as hypertension and diabetes due to its analgesic and diuretic properties (Bueno et al. 2021). Therefore, a comprehensive evaluation of REE concentrations in *B. trimera* and its surrounding environment (soil and surface waters) is essential to trace the migration of these elements from carbonatite rocks. Nevertheless, the use of a single plant species may reflect specific functional traits of the species, and further research is required to understand the transfer mechanisms of REEs across different vegetation types.

In this context, this study aimed to establish a correlation between REE concentrations in soils and plants near carbonatite outcrops in southern Brazil. The objectives were to evaluate REE distribution and fractionation in soils at different distances from carbonatite outcrops, to assess REE mobility (accumulation and translocation) in *B. trimera* roots and leaves, and to determine whether lithological geochemical signatures remain constant across rock, soil, and plant components. Thus, we sought to integrate geochemical and biological data in order to provide a more comprehensive understanding of the role of soils and plants in mediating REE mobility in carbonatite-influenced ecosystems.

## Materials and methods

### Study area and sample collection

**Fig. 1:**
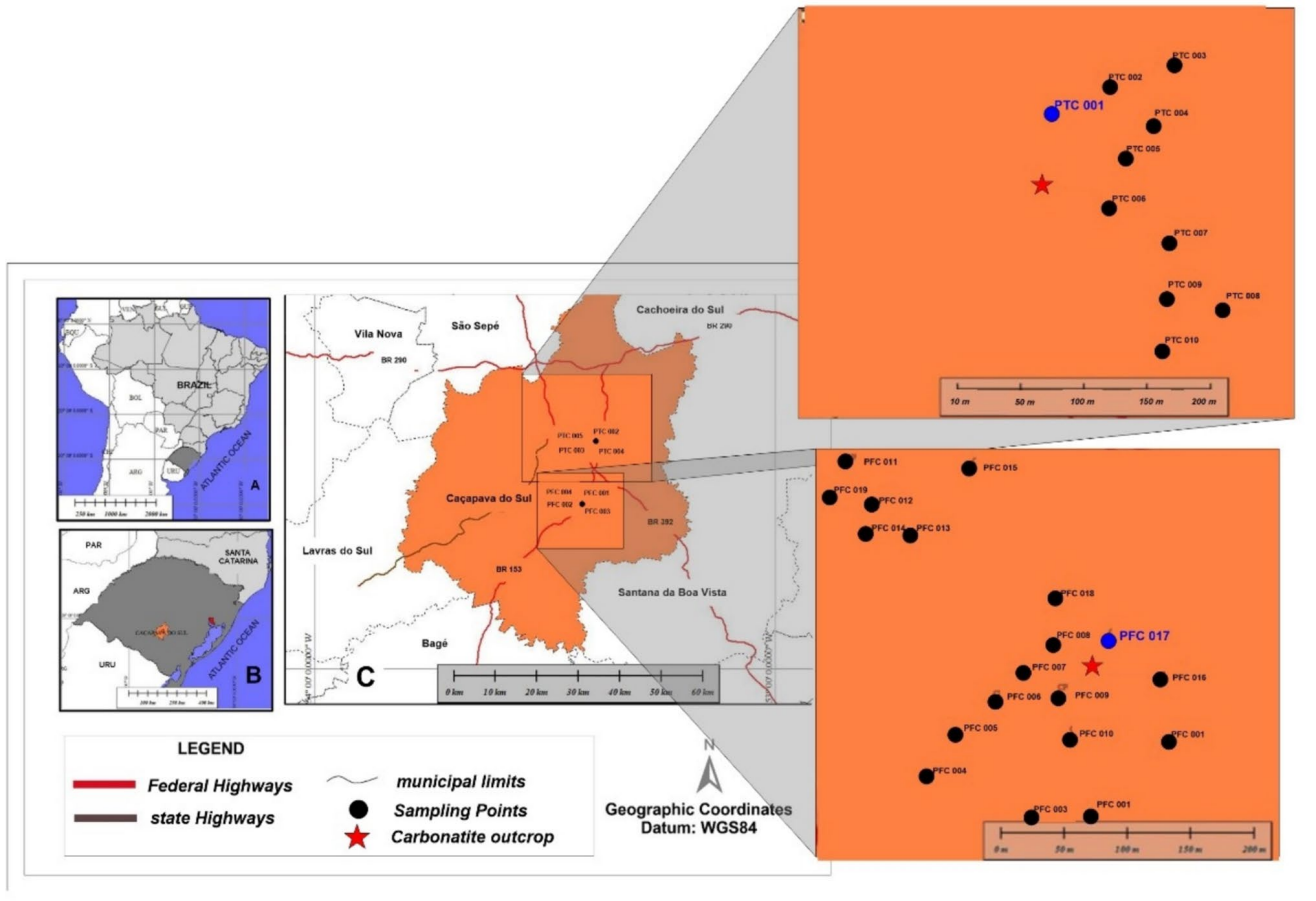
Map of the study areas; the red stars indicate carbonatite outcrops, PTC above and PFC below. The black dots demonstrate the collection points for soil, root, and leaf samples of *B. trimera*; the blue dots are the samples that showed the highest concentration of REEs

This study was conducted in the municipality of Caçapava do Sul in central Rio Grande do Sul. Two areas (illustrated in Fig. 1) exhibiting carbonatite occurrences were selected on the basis of the geological surveys conducted by Cerva-Alves et al. (2017) and Morales et al. (2019). The first area (267,488.81 E and 6,607,148.93 S) corresponds to the Passo Feio carbonatite (PFC), covering approximately 30.6 m^2^, and the second area (269,556.69 E and 6,620,279.09 S), designated Picada dos Tocos carbonatite (PTC), has a sampling area of around 10.5 m^2^. The coordinates were recorded using the WGS 84 geodetic reference system in UTM Zone 22 J.

The predominant soil classes in the Caçapava do Sul area are Cambisoils and Litholic Neosoils (Santos et al. 2018). These soil types are characterized by their shallow depth and heterogeneous mineralogy, which are directly influenced by the source material. In regions where carbonatitic outcrops are more prevalent, soils characteristically exhibit elevated carbonate content and pH variability, particularly in environments with minimal leaching (Brioschi et al. 2013). The combination of these edaphic characteristics with the region’s humid subtropical climate, characterized by annual rainfall of 1300–1500 mm, fosters active weathering processes and contributes to the redistribution and potential mobility of REEs within the soil profile (Inmet 2025).

Soil and *B. trimera* root and leaf samples were collected in February 2025 from both areas surrounding the carbonatite outcrops. The sampling points were selected based on the distribution of individuals of the plant species within the delineated area. In total, 30 samples (10 from soil, 10 from leaves, and 10 from roots) were collected from the PTC region and 53 (19 from soil, 19 from leaves, and 15 from roots) were acquired from the PFC region. Each sample was georeferenced individually and assigned an identification code. Soil samples were collected at a depth of 0–20 cm, which was immediately below where the *B. trimera* roots and leaves were collected. Samples of both carbonatites were also collected in order to compare them to the soil and vegetation samples.

### Sample preparation

*B. trimera* samples were initially separated into roots and leaves, washed with distilled water to remove surface residues, dried in an oven at 40 °C for 72 h, and then ground in an analytical mill (Q298A, Quimis, Brazil). The resulting plant material was then sieved to obtain ≤ 1-mm particles. The soil samples were subjected to drying in an oven at 40 °C for 72 h. Each soil sample was then separated into two distinct fractions: the first fraction contained < 2-mm particles and was utilized for physicochemical characterization, whereas the second was ground and sieved until < 177-µm particles were obtained, which were then used to determine the concentrations of REEs and other elements. All prepared samples were stored in polyethylene bottles protected from light and humidity until the analysis.

### Analytical methods

Soil samples (< 2-mm particles) were submitted to a pH analysis in a 0.1 M KCl solution (with a soil-to-solution ratio of 1:2.5), and the pH was measured using a pH meter (Model One Sense pH 2500, Marte Cientifica, Brazil) (Landim et al. 2022). Raman spectroscopy was performed using a Bruker Senterra confocal Raman microscope with a 532-nm laser operating in the spectral range of 3500–50 cm^‒1^ for the mineralogical analysis. X-ray diffraction (XRD) analyses were performed using a Miniflex® 300 diffractometer (Rigaku, Japan) equipped with Cu Kα radiation (*λ* = 1.54051 Å), operating at 30 kV and 10 mA. Diffractograms were recorded over a 2θ range of 5–100°. Crystalline phases were identified by comparison with reference patterns available in the RRUFF database (Lafuente et al. 2015).

The REE concentrations were determined at the Activation Laboratory in Ontario (Canada) using inductively coupled plasma mass spectrometry (ICP-MS). The samples were previously prepared for further analysis by ICP-MS. The sample preparation methodology is described as follows. The soil analysis procedure required 0.25 g of each sample to be digested with four acids. The samples were ground and granulometrically separated into 0.177-µm particles, initially digested with fluoridric acid, subsequently with a mixture of nitric acid, perchloric acid, and aqua regia. In order to analyze the *B. trimera* root and leaf samples, the samples (1 mm) were digested in aqua regia at 95 °C for 2 h.

### Calculations

To investigate the potential geochemical fractionation of REEs in soil samples, Ce and Eu anomalies were calculated using concentrations that were normalized to the upper continental crust (UCC). These calculations were based on the reference values established by Taylor and McLennan (1995). The calculation of the anomalies was performed using Eqs. 1 and 2 (Brioschi et al. 2013):

1 $$\frac{\mathrm{C}\mathrm{e}}{{\mathrm{C}\mathrm{e}}^{*}}=\frac{{3\mathrm{C}\mathrm{e}}_{\mathrm{n}\mathrm{o}\mathrm{r}\mathrm{m}}}{{2\mathrm{L}\mathrm{a}}_{\mathrm{n}\mathrm{o}\mathrm{r}\mathrm{m}}+{\mathrm{N}\mathrm{d}}_{\mathrm{n}\mathrm{o}\mathrm{r}\mathrm{m}}}$$

2 $$\frac{\mathrm{E}\mathrm{u}}{{\mathrm{Eu}}^{*}}=\frac{{\mathrm{Eu}}_{\mathrm{n}\mathrm{o}\mathrm{r}\mathrm{m}}}{\sqrt{\left({\mathrm{Sm}}_{\mathrm{n}\mathrm{o}\mathrm{r}\mathrm{m}}*{\mathrm{Gd}}_{\mathrm{n}\mathrm{o}\mathrm{r}\mathrm{m}}\right)}}$$

where Ce_norm_, Eu_norm_, La_norm_, Nd_norm_, and Gd_norm_ are the concentrations of each element normalized to the UCC. The bioaccumulation factor (BAF) was determined to evaluate plant roots’ ability to absorb REEs from the soil using Eq. 3, whereas the translocation factor (TF) was determined to assess the mobility of REEs from the roots to the aerial parts (leaves) using Eq. 4 (Khan et al. 2017):

3 $$\mathrm{B}\mathrm{A}\mathrm{F}=\frac{{\left[\mathrm{R}\mathrm{E}\mathrm{E}\right]}_{\mathrm{r}\mathrm{o}\mathrm{o}\mathrm{t}}}{{\left[\mathrm{R}\mathrm{E}\mathrm{E}\right]}_{\mathrm{s}\mathrm{o}\mathrm{i}\mathrm{l}}}$$

4 $$\mathrm{T}\mathrm{F}=\frac{{\left[\mathrm{R}\mathrm{E}\mathrm{E}\right]}_{\mathrm{l}\mathrm{e}\mathrm{a}\mathrm{f}}}{{\left[\mathrm{R}\mathrm{E}\mathrm{E}\right]}_{\mathrm{r}\mathrm{o}\mathrm{o}\mathrm{t}}}$$

where [REE]_root_ is the total concentration of a given REEs in root tissue, [REE]_soil_ is its corresponding total concentration in the soil, and [REE]_leaf_ is the concentration of the REEs in the leaf tissue.

### Statistical analysis

The subsequent calculation of the descriptive statistics and normalization was conducted utilizing Microsoft Excel software. Pearson’s correlation matrices were generated using the R software (v, 4.5.0), with the Corrplot function from the GGally package employed to construct the correlation matrix. Principal component analysis (PCA) was performed in R software (v. 4.5.0) using the FactoMineR, Factoextra, and readr packages. A correlation matrix and *z*-score normalization were used to minimize differences in data magnitude.

## Results and discussion

### REEs in soil samples

The descriptive values of REE concentrations in the soil samples are listed in Table 1. The soils in proximity to the PFC and PTC outcrops exhibited notable enrichment in REEs, particularly La, Ce, and Nd. This observation is supported by the elevated means and medians of these elements, indicating a trend in the data. The distribution between LREEs and HREEs demonstrates a predominance of LREEs in the analyzed samples, accounting for 89.71% of the mean of ΣREEs.

**Table 1:** Mean, minimum, and maximum REE concentrations (mg kg^‒1^) in soil samples from the PFC and PTC areas

REEs	Soil samples (*n* = 29)		
	Minimum	Maximum	Mean
La	39.9	426	180
Ce	95.3	984	404.5
Pr	11.8	117	48.7
Nd	44.9	418	177.8
Sm	9	62	30.4
Eu	1.76	19	8.22
Gd	7.1	49.9	22.8
Dy	3.60	26.4	12.5
Tb	0.9	5.9	2.71
Ho	0.5	4.2	2.03
Er	1.2	10.3	5.13
Tm	0.20	1.2	0.645
Yb	0.9	6	3.3
Lu	0.1	0.9	0.486
Y	11.1	101	47.8
ΣREEs	240.1	2231.8	947
ΣLREEs	211.5	2026.0	849.6
ΣHREEs	26.4	205.8	97.3
ΣLREEs/ΣHREEs	4.37	12.55	8.41
Ce/Ce^*^	0.835	1.13	1.01
Eu/Eu^*^	0.87	1.48	1.27

Ce/Ce^*^, cerium anomaly; Eu/Eu^*^, europium anomaly.

A comparative analysis reveals that soils from the PFC and PTC regions exhibit higher mean concentrations of ΣREEs than those observed in other areas of Brazil and worldwide. Landim et al. (2022) studied soil samples from Piauí State (northeastern Brazil) and reported a mean ΣREE concentration of 107.8 mg kg^‒1^, which is approximately nine times lower than those recorded in areas associated with carbonatites (Landim et al. 2022). Compared to soils from other countries, the mean ΣREE values in the PFC and PTC regions are about 12.7 times higher than those reported for Cuba and 6.1 times higher than those for China (Alfaro et al. 2018; Wei et al. 1991). A comparative analysis with other regions is illustrated in Supplementary Fig. S1.

Comparing the REE concentrations observed in this study with those measured in soils adjacent to carbonatites reveals similar results. Brioschi et al. (2013) investigated REE concentrations in soils developed over a carbonatite body in Breisgau (Germany) and reported higher mean concentrations for La, Ce, and Nd, with values of 313, 499, and 146 mg kg^‒1^, respectively. The mean total concentration of ΣLREEs was 1079 mg kg^‒1^, representing approximately 95% of the ΣREEs. This finding reveals a marked predominance of LREEs (Brioschi et al. 2013). The similarity in REE distribution and enrichment patterns between the soils of Breisgau and those adjacent to the PTC and PFC regions reinforces the decisive role of the parent rock in the geochemistry of soils developed from these lithotypes. This influence is also observed in the higher REE concentrations in samples PFC017S and PTC001S (Supplementary Table S1), which, being closer to the carbonatite outcrops, contain 2231.8 and 1692.4 mg kg^‒1^ of ΣREEs, respectively. In contrast, samples from areas farther from the outcrops of both carbonatites showed lower ΣREE values: PFC019S (240.1 mg kg^‒1^) and PTC003S (215.4 mg kg^‒1^).

**Fig. 2:**
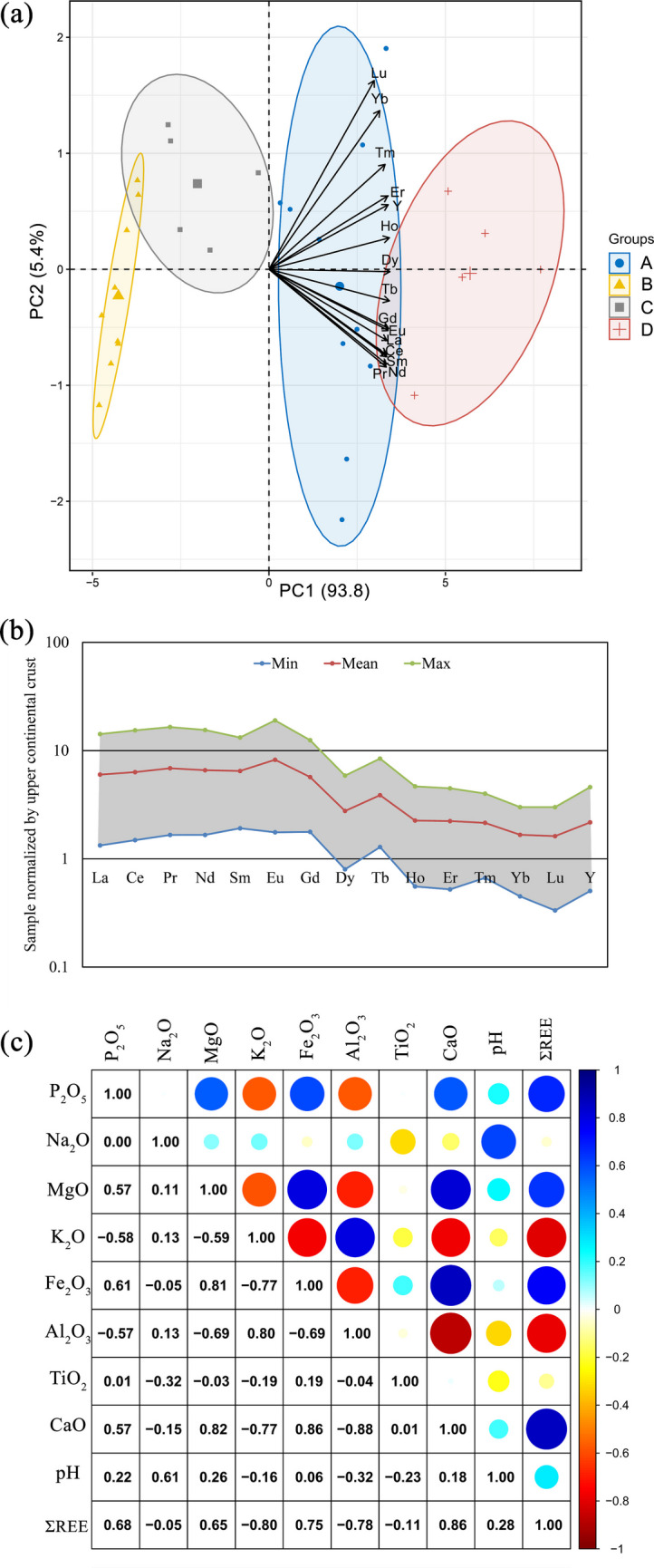
PCA with soil samples (**a**); REE concentrations (mg kg.^‒1^) normalized by the upper continental crust as per Taylor and McLennan (1995). Values above 1 reveal enrichment and below confirm depletion (**b**); Pearson’s correlation matrix of the variables is presented in Supplementary Table S3; the circle size indicates the amplitude, and the color indicates the correlation values (**c**)

A PCA using individual REE concentrations as variables was employed to better understand the distribution of soil samples and the influence of carbonatite outcrops in the study areas (Table S1). The PCA revealed a strongly structured multivariate system, in which the first two PCs explained 99.2% of the total variance (Fig. 2a). This high value supports strong covariance between REE concentrations and demonstrates that their distribution is governed by a limited number of dominant geochemical controls. Under these conditions, the PCA projection preserved virtually the entire multivariate structure of the dataset, allowing for a robust interpretation of REE behavior with minimal loss of information. The ellipses associated with each group were constructed at a confidence level of 0.75 to emphasize central compositional trends and minimize the influence of extreme values.

Furthermore, the PCA identified four compositional groups, with samples from the PFC and PTC areas distributed across all of them. The absence of distinct clustering in the PCA revealed that the differences between PFC and PTC are not the main axis of variability in the system. Instead, geochemical controls related to the degree of weathering and proximity to outcrops are more decisive, resulting in gradual transitions between groups rather than well-defined geochemical boundaries. Table S2 presents all the samples that comprise the groups.

The spatial distribution of samples relative to the carbonatite outcrops further provides the geochemical controls operating in the system. Samples from group D, distinguished by their elevated REE concentrations (Table S1), were predominantly located in areas near the outcrops (Fig. 1). This supports a significant and direct influence of carbonatite on the soils’ geochemical composition. Conversely, samples from group B, which exhibited the lowest REE concentrations, were predominantly associated with regions farther from the outcrops, where the direct influence of the carbonatite body was diminished. This spatial pattern confirms a progressive decrease in carbonatite contribution as distance from the outcrops increases, possibly representing an indirect indication of the lateral extent and geochemical influence of the subsurface carbonatite body.

The degree of weathering of the samples was assessed using the Al_2_O_3_/CaO ratio, as proposed by Chen et al. (2023), whose values are presented in Table S3. While this ratio was not employed as an input variable in the PCA, it offers an independent line of evidence for interpreting the variability of REE concentrations in the study areas. Groups exhibiting elevated REE concentrations, specifically group D and to a lesser extent group A, exhibited reduced Al_2_O_3_/CaO ratios. This demonstrates that these groups experienced enhanced preservation of calcium-rich phases, which are known to frequently host REEs such as carbonates and apatite (Guo et al. 2024). The limited calcium removal in these groups reveals restricted chemical alteration, allowing REEs to remain associated with primary or slightly altered mineral assemblages (Siegfried et al. 2025). Conversely, group B, which exhibited the lowest REE concentrations, is associated with markedly higher and more variable Al_2_O_3_/CaO ratios. These ratios reflect intense calcium leaching during advanced stages of soil weathering (Tyler 2004). The preferential removal of calcium-rich minerals in this group likely results in dilution, redistribution, or partial loss of carbonatite-derived REE signature, leading to lower overall concentrations of these elements.

Comparing PFC and PTC soil samples with those from the UCC revealed significant REE enrichment, particularly LREEs. This pattern is also illustrated in Fig. 1B, where lines above 1 confirm REE enrichment and lines below 1 highlight depletion. Notably, the Ce and Eu anomalies showed mean values of 1.01 and 1.27 (Table 1), respectively. Conventionally, values above 1 demonstrate positive anomalies, while values below 1 represent negative anomalies. The Ce anomaly value, being near 1, does not reveal a significant anomaly. The slight positive anomaly for Eu supports preservation of the geochemical signature of the parent rock, indicating mild weathering conditions or low mobility. Both values are consistent with those found for soils developed over carbonatites (Brioschi et al. 2013).

Figure 2c shows Pearson’s correlation matrix between major elements, ΣREEs, and soil pH. The positive correlations with CaO (*r* = 0.86) and P_2_O_5_ (*r* = 0.68) reinforce the hypothesis that carbonatite geochemistry, characterized by its richness in carbonates and phosphates, is a contributing factor (Anenburg and Walters 2024; Xue et al. 2020). The positive correlation between ΣREEs and Fe_2_O_3_ (0.75) confirms that iron oxides play an important role in REE retention. Previous research has demonstrated that secondary Fe minerals (e.g., goethite and hematite) possess reactive surfaces that facilitate REE adsorption, particularly in less acidic soils (Sager and Wiche 2024; Zhang et al. 1998). This observation is consistent with the pH values recorded and the positive correlation with pH (0.29), indicating that less acidic environments promote surface retention of REE oxides. The substantial correlation with MgO (0.65) underscores the association with the carbonatitic origin, as dolomite is a prevalent constituent in these structures (Morales et al. 2019). This link may also highlight the presence of forms of Mg that can co-precipitate or adsorb REEs. Conversely, the negative correlation with Al_2_O_3_ (− 0.78) suggests a lack of association between REEs and aluminosilicate phases (Coppin et al. 2002). Furthermore, the negative correlation between ΣREEs and K_2_O (− 0.80) demonstrates that soils with high REE concentrations tend to have low K levels. This finding supports the hypothesis that mineralogical units rich in Ca, Mg, and Fe also serve as hosts for REEs. Additionally, K mobility due to weathering can be substantial, hindering its retention (Ingrid 1998; Schmidt et al. 2024).

The mineralogical compositions of PFC and PTC carbonatite bodies contained accessory REE-bearing minerals characteristic of carbonatite rocks, including monazite-(Ce), fluorapatite, aeschynite-(Ce), pyrochlore, and bastnaesite (Morales et al. 2019; Remus et al. 2000; Vieira et al. 2025). Soil sample PFC017S exhibited higher REE concentrations compared to the other samples analyzed (Supplementary Table S1). To identify the source of this enrichment, a mineralogical analysis was performed, incorporating XRD and Raman spectroscopy. The obtained diffractograms and spectra exhibit characteristic peaks of quartz, illite, and apatite in the XRD (Supplementary Fig. S2), suggesting a weathered soil matrix with phosphate phases that potentially host REEs. The Raman spectrum confirmed the presence of fluorapatite and monazite, which are classic rare earth-bearing minerals (Song et al. 2023). In addition, complex oxides from the polycrasite/aeschinite family were detected, which are also recognized as REE hosts in crustal environments (Guastoni et al. 2019). These minerals are commonly reported in carbonatitic rocks, further reinforcing the relationship between the lithology of origin and the anomalous REE concentrations observed in this soil, associated with both primary phosphates and complex oxides (Cerva-Alves et al. 2017; Vieira et al. 2025).

### REEs in vegetation samples

REEs are not essential nutrients for plants, although they can be absorbed from soil and rocks that are rich in these elements (Balaram 2019). The concentration of these compounds in plants is typically low but may vary depending on the species and growth environment. Once absorbed, these compounds are transported through the xylem to other tissues.

Descriptive values of REE concentrations in the *B. trimera* roots and leaves are listed in Table 2. The results indicated substantial REE concentrations, with mean ΣREE values of 25.9 mg kg^‒1^ in the roots and 1.86 mg kg^‒1^ in the leaves. These data demonstrate a pattern of preferential accumulation in the roots, consistent with other studies involving plants exposed to metals (Zadokar et al. 2023). In addition, the mean LREE concentrations were comparatively higher than that of HREEs, with Ce, La, and Nd showing the most significant prevalence, which is attributable to their higher inherent abundance (Dyakova 2023). The REE concentrations in the roots and leaves are consistent with those observed in plants tolerant to these elements, which vary by 0.04–950 mg kg^‒1^ for LREEs (Ashraf et al. 2022). Nevertheless, the concentrations remained at levels above those reported elsewhere (Brioschi et al. 2013; Dyakova 2023). Supplementary Table S4 presents the concentration values for each root and leaf sample.

**Table 2:** The mean, minimum, and maximum REE concentrations (mg kg^‒1^) in B. trimera root and leaf samples from the PFC and PTC areas. Ce/Ce^*^, cerium anomaly; Eu/Eu^*^, europium anomaly

REEs	Roots			Leaf		
	Min	Max	Mean	Min	Max	Mean
La	0.750	19.9	5.99	0.130	2.11	0.484
Ce	1.38	36.1	10.3	0.188	3.78	0.627
Pr	0.155	4.27	1.25	0.0230	0.428	0.091
Nd	0.593	16.5	4.85	0.0890	1.66	0.356
Sm	0.101	2.34	0.710	0.0160	0.301	0.062
Eu	0.021	0.586	0.177	0.00260	0.0537	0.012
Gd	0.072	1.61	0.529	0.0101	0.159	0.039
Dy	0.044	0.862	0.311	0.00570	0.0944	0.023
Tb	0.009	0.163	0.0557	0.000900	0.0162	0.00396
Ho	0.007	0.134	0.0509	0.00110	0.0145	0.00391
Er	0.0204	0.318	0.128	0.00220	0.0351	0.00955
Tm	0.00260	0.0377	0.0153	0.000200	0.00460	0.00117
Yb	0.0143	0.238	0.0924	0.00160	0.0242	0.00653
Lu	0.00180	0.0284	0.0120	> LoD	0.00340	0.000766
Y	0.202	3.43	1.36	0.039	0.393	0.135
ΣREEs	3.37	86.3	25.9	0.528	9.00	1.86
ΣLREEs	3.00	79.7	23.3	0.0251	8.25	1.43
ΣHREEs	0.373	6.64	2.55	0.0616	0.744	0.224
ΣLREEs/ΣHREEs	0.00394	0.0133	0.00824	0.000359	0.0111	0.00630

REE absorption by roots can occur through passive and active mechanisms involving poorly selective ion transporters, especially those intended for Ca^2^⁺ and Mg^2^⁺. Typically, REEs are present in a trivalent form (REE^3+^), with an ionic radius similar to that of Ca^2+^. This similarity allows them to be absorbed by transporters such as calcium channels through cation exchange systems (Sager and Wiche 2024). Research has further demonstrated that REEs can bind to organic acids exuded by roots, including citrate and malate. This binding leads to the formation of soluble complexes, which enhance bioavailability and facilitate entry into plant cells. Upon reaching root tissue, REEs tend to accumulate in the apoplast or become sequestered in vacuoles. This adaptation restricts their translocation to upper tissues (Tao et al. 2022).

The reduced REE concentrations in leaves can be attributed to various physiological and biochemical barriers. One primary mechanism involves the endodermal barrier of the root, where suberin and lignin in the Casparian strip act as a selective physicochemical filter, restricting the movement of metal ions to the xylem (Barberon and Geldner 2014; Dell’Aglio 2021). Even REEs that reach the xylem experience translocation difficulties due to the low mobility of trivalent elements in raw sap (Poschenrieder et al. 2019). Additionally, REEs can form insoluble complexes with phosphates, pectins, and other compounds present in cell walls, contributing to their fixation in roots (Ding et al. 2005; Guo et al. 2023). This behavior may indicate a strategy to tolerate potentially toxic environmental elements. By retaining REEs in the roots, plants avoid accumulation in photosynthetic tissues, which could interfere with essential metabolic processes such as photosynthesis, pigment synthesis, and nitrogen metabolism (Tao et al. 2022).

**Fig. 3:**
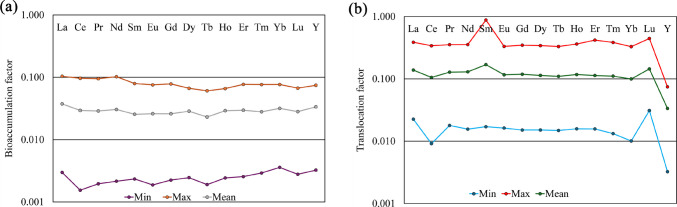
BAF and TF values of the REEs determined

The BAF (Eq. 3) was utilized to assess the ability of roots to absorb and accumulate REEs, and BAF values above 1 indicated high accumulation capacity, while values below 1 suggested low absorption or exclusion by the plant. The TF (Eq. 4) is a metric used to evaluate the efficiency of REE transport from the roots to the aerial tissues. TF values above 1 indicated high internal mobility and effective redistribution of the element within the plant. Conversely, values below 1 suggested preferential accumulation in roots, with reduced translocation to aerial parts (Khan et al. 2017). Moreover, we noted a discernible trend in both BAF (Fig. 3a) and TF (Fig. 3b). The BAF values ranged from 0.006 to 0.083, indicating low absorption efficiency of REEs relative to their availability in the soil, whereas TF ranged from 0.03 to 0.88, confirming preferential REE retention in the underground parts of *B. trimera*. The calculated values are presented in Supplementary Tables S5 and S6. The calculated Ce and Eu anomalies demonstrated a negative Ce anomaly, with mean values of 0.818 and 0.570 for the roots and leaves, respectively. A positive Eu anomaly was also observed, with mean values of 1.51 and 1.40 for the roots and leaves, respectively. These anomalies are consistent with previous studies on REE concentrations in vegetation and support the hypothesis of low Ce mobility from the soil to the *B. trimera* roots and leaves (Brioschi et al. 2013; Fu et al. 2001; Pourret et al. 2022). Nevertheless, the positive Eu anomaly was also observed in soil samples, illustrating the environmental influence on REE concentrations.

**Fig. 4:**
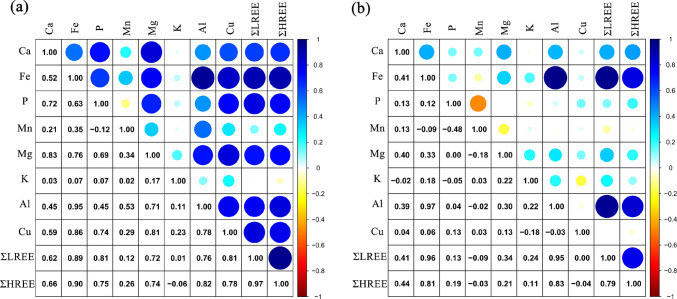
Pearson’s correlation matrix for the macronutrients, micronutrients, ΣLREEs, and ΣHREEs for *B. trimera* roots (**a**) and leaves (**b**). The values utilized are listed in Supplementary Table S7

Figure 4a presents Pearson’s correlation matrix, which illustrates the interrelationships among LREEs, HREEs, micronutrients, and macronutrients in *B. trimera* roots*.* Positive correlations were observed between the concentration of both ΣLREEs and ΣHREEs and the contents of Fe, Al, Mg, Ca, and Cu in root tissues. This pattern suggests co-retention mechanisms rather than active co-uptake, in which REEs and major cations are immobilized in the rhizosphere and root tissues through adsorption onto cell walls, association with Fe- and Al-rich oxide phases, and sequestration in apoplast compartments. It has been demonstrated in previous studies that Fe-and Al-oxyhydroxides offer reactive surfaces that efficiently retain REEs through surface complexation and ion exchange processes (Aubert et al. 2001). The positive correlation with P further supports the preferential accumulation of REEs in roots relative to leaves, as REEs are known to form stable complexes with phosphate groups within cellular compartments, which restrict their mobility and limit upward translocation, thereby reducing potential phytotoxic effects (Tao et al. 2022). Such root-dominated retention of REEs has been widely reported as a common feature in soil–plant systems, particularly in metal-rich environments (Tyler 2004).

In contrast, a significant positive correlation was observed only between REEs and Al in *B. trimera* leaves (Fig. 4b), indicating a distinct behavior in elemental redistribution within aerial tissues. The association with Al suggests selective translocation processes, potentially involving REE-Al complexes stabilized by low-molecular-weight organic acids such as citrate and malate, which enhance solubility and facilitate transport in the xylem (Ma 2000; Yuan et al. 2017). During internal transport, Fe levels are strictly regulated, with most being retained in the roots. Conversely, REEs have been observed to maintain mobility as organic or Al-associated complexes, thereby explaining the absence of a correlation between REEs and Fe concentrations in leaves (Yuan et al. 2017).

The lack of significant correlations between REEs and other elements in leaves, together with low TF values, indicates strong physiological filtering and selective control of metal entry into photosynthetic tissues. Taken together, these observations suggest that *B. trimera* exhibits an excluder-like behavior with respect to REEs, in which metals are efficiently immobilized in roots through geochemical and physiological barriers. At the same time, only a limited and chemically selected fraction is translocated to aerial tissues. Such selective translocation and retention patterns have been reported to vary among plant species, reflecting species-specific tolerance strategies and differences in ligand production and internal metal handling (Zhang et al. 2019; Sommer et al. 2023).

### Relationships of REE concentrations in the bedrock-soil-root-leaf system

**Fig. 5:**
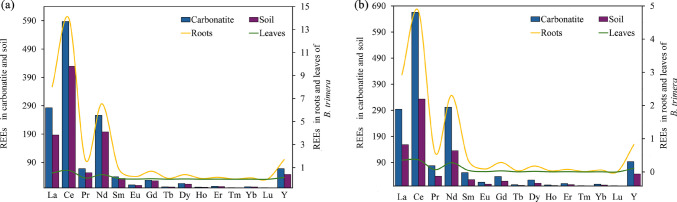
The relationship of REE concentrations (mg kg.^‒1^) of carbonatite rock, soil, and *B. trimera* roots and leaves of for the PFC area (**a**) and PTC area (**b**)

The mean REE concentrations in the system composed of carbonatite rock, soil, and *B. trimera* roots and leaves of in both study areas are presented in Fig. 5. Representative rocks were also analyzed for total REE concentrations (Supplementary Table S8). A consistent pattern of enrichment of LREEs relative to HREEs was observed across all investigated matrices. Among the LREEs, Ce exhibited the highest mean concentration, followed by La and Nd, which corroborates the prevailing geochemical characteristics of carbonatite systems (Chakhmouradian and Zaitsev 2012). A comparative analysis of the HREEs also revealed that Gd, Dy, and Y exhibited comparatively higher mean concentrations, albeit still lower than those observed for the LREEs.

The anomalies calculated for Ce and Eu reinforce this pattern. In both study areas, carbonatite rocks exhibited no significant Ce anomalies, with values approaching 1, indicating the absence of relevant fractionation for this element (Supplementary Table S8). Conversely, a positive Eu anomaly was observed, suggesting the preservation of mineral phases rich in Eu^2^⁺ or the inheritance of the magmatic signature of the carbonatite body. Analogous outcomes were obtained for soils, suggesting that active weathering maintained the fundamental geochemical imprint of carbonatites, with only negligible alterations throughout the system.

This distribution pattern was confirmed by Pearson’s correlation coefficients calculated between the mean concentrations of REEs in the different matrices, which yielded *r* > 0.92 (Supplementary Table S8). The results indicated a strong positive correlation, suggesting that the geochemical signature observed in soils and plant tissues is strongly controlled by the composition of the carbonatite parent rock (Brioschi et al. 2013).

Another notable point concerns the observed fractionation accompanied by LREE enrichment, which is consistent with moderate weathering processes in carbonatite-bearing soils, as LREEs exhibit a stronger tendency to bind with secondary phases such as iron oxides and phosphates (Landim et al. 2022). Conversely, HREEs demonstrate greater relative mobility in solution, particularly in environments favorable to organic complexation. This phenomenon may explain the persistence of the LREE signature even after these elements have transitioned from rock to soil and subsequently to plants (Tyler 2004). The concentrations observed in the analyzed plants exhibited a similar trend to those in the soil, indicating that root absorption was not selective enough to alter the pattern inherited from the substrate. Research on plant species indicates that, although the BAF for REEs is typically low, the relative transfer between LREEs and HREEs tends to maintain the characteristic imprint of lithogenic origin (Sager and Wiche 2024). The results presented herein confirm the strong influence of carbonatites on the geochemistry of local soils and vegetation, reinforcing the importance of these igneous bodies as a source of REE enrichment in the environment.

The PCA integrating REE concentrations from carbonatite rocks, soils, roots, and leaves highlights the hierarchical structure of the bedrock-soil–plant system (Fig. S3). The PC1 accounted for 95.90% of the total variance, reflecting the predominant lithological influence of the carbonatite parent material. Rocks and soils delineated the primary geochemical gradient, while plant tissues were distributed along a similar compositional framework. The PC2 accounted for 3.33% of the variance, capturing subtler fractionation within the REE series and separating a subset of leaf samples from the remaining compartments. This phenomenon aligns with the observed correlation results, which indicate the co-retention of REEs with Fe, Al, Ca, Mg, and P in root tissue, in contrast to the selective associations observed in leaf tissue and the low translocation factors that were measured. Collectively, these findings suggest that while the distribution of elements in roots (i.e., REEs) was predominantly influenced by lithology and pedogenic inheritance, physiological filtration during root uptake and internal transport exerted a secondary control on the redistribution of elements within the plant tissues.

### Environmental implications of REE concentrations

REE concentrations in soil samples exhibit higher levels than the average concentrations reported in the Earth’s crust (Sager and Wiche 2024). Nevertheless, these concentrations remain consistent with those reported in the literature for carbonatite-derived systems (Brioschi et al. 2013). The predominance of LREEs, the absence of a significant Ce anomaly, and the low bioaccumulation and translocation factors suggest that pedogenic processes in the studied system favor retention, resulting in limited mobility and reduced bioavailability. No significant transfer to aerial plant tissues was observed, indicating that the REEs were predominantly retained in the *B. trimera* roots, limiting their translocation through the plant.

Hence, the elevated REE concentrations in soils were consistent with natural enrichment in carbonatite-derived systems and did not indicate anthropogenic contamination. In addition, the restricted mobility and limited transfer to aerial tissues suggest that REEs were largely retained within the soil-root compartment, potentially minimizing impacts on soil functioning and trophic interactions under natural conditions.

## Conclusion

This study demonstrated that carbonatite outcrops in Caçapava do Sul (southern Brazil) exert a strong control on the distribution of REEs in surrounding soils and vegetation. Soils developed over the Passo Feio and Picada dos Tocos carbonatites showed significant REE enrichment, particularly in LREEs, reflecting the mineralogical composition and geochemical signature of the parent rocks.

*Baccharis trimera* exhibited preferential accumulation of REEs in roots, while concentrations in leaves remained considerably lower. The low bioaccumulation and translocation factors indicate restricted mobility of REEs within the plant and suggest that root tissues act as an effective barrier restricting transfer to aerial compartments.

Strong correlations between rock, soil, and plant compartments confirmed that carbonatite lithology is the main controlling factor governing the distribution of REEs in the studied system. In addition, the gradual decrease in ΣREE concentrations with increasing distance from the outcrops highlights the potential application of soil geochemistry as a proxy for identifying carbonatite-influenced areas.

Overall, the results indicate that the elevated REE concentrations observed in the study area are predominantly geogenic rather than anthropogenic and that REE mobility remains limited under the investigated environmental conditions. These findings contribute to a better understanding of REE dynamics in subtropical carbonatite systems and provide useful information for environmental assessment and geochemical exploration studies.

## Supplementary Information

Below is the link to the electronic supplementary material.

ESM 1 (DOCX 4.81 MB)

## Data Availability

Data will be made available on request.
