# Supplementary material for: The dynamics of rare earth elements in soil–plant systems in southern Brazil: a case study of carbonatite-bearing areas and Baccharis trimera
Source: Environ Sci Pollut Res Int. 2026 Jun 11;33(19):9670–82. doi: 10.1007/s11356-026-37896-5 (PMC13294312; doi:10.1007/s11356-026-37896-5)
Supplement: Supplementary file 1 — (DOCX 4.81 MB) [file 11356_2026_37896_MOESM1_ESM.docx]

**Supplementary Information**

**The dynamics of rare earth elements in soil-plant systems in southern Brazil: A case study of carbonatite-bearing areas and *Baccharis trimera***

Lucas Mironuk Frescura^a,b^ (ORCID 0000-0002-7906-0254) · Nicole Werle da Silva^a,c^ (ORCID 0009-0009-6766-0963) · Daniel Triboli Vieira^d^ (ORCID 0000-0003-0616-5407) · Maria Luiza de Vargas Mallmann^a^ (ORCID 0009-0008-8467-4980) · Edinei Koester^d^ (ORCID 0000-0002-4424-4782) · Rogerio Vescia Lourega^a^ (ORCID 0000-0001-8154-0524) · Marcelo Barcellos da Rosa^b,c^ (ORCID 0000-0001-5959-0381)

^a^ Chemical and Pharmaceutical Research Laboratory, Universidade Federal de Santa Maria, Brazil

^b^ Post-Graduate Program in Pharmaceutical Sciences, Universidade Federal de Santa Maria, Santa Maria, RS, Brazil

^c^ Post-Graduate Program in Chemistry, Universidade Federal de Santa Maria, Santa Maria, RS, Brazil

^d^ Geosciences Institute, Universidade Federal do Rio Grande do Sul, Porto Alegre, RS, Brazil

Corresponding author: Marcelo Barcellos da Rosa

Email: [marcelo.b.rosa@ufsm.br](mailto:marcelo.b.rosa@ufsm.br)

Phone: +55-55-3220 8066


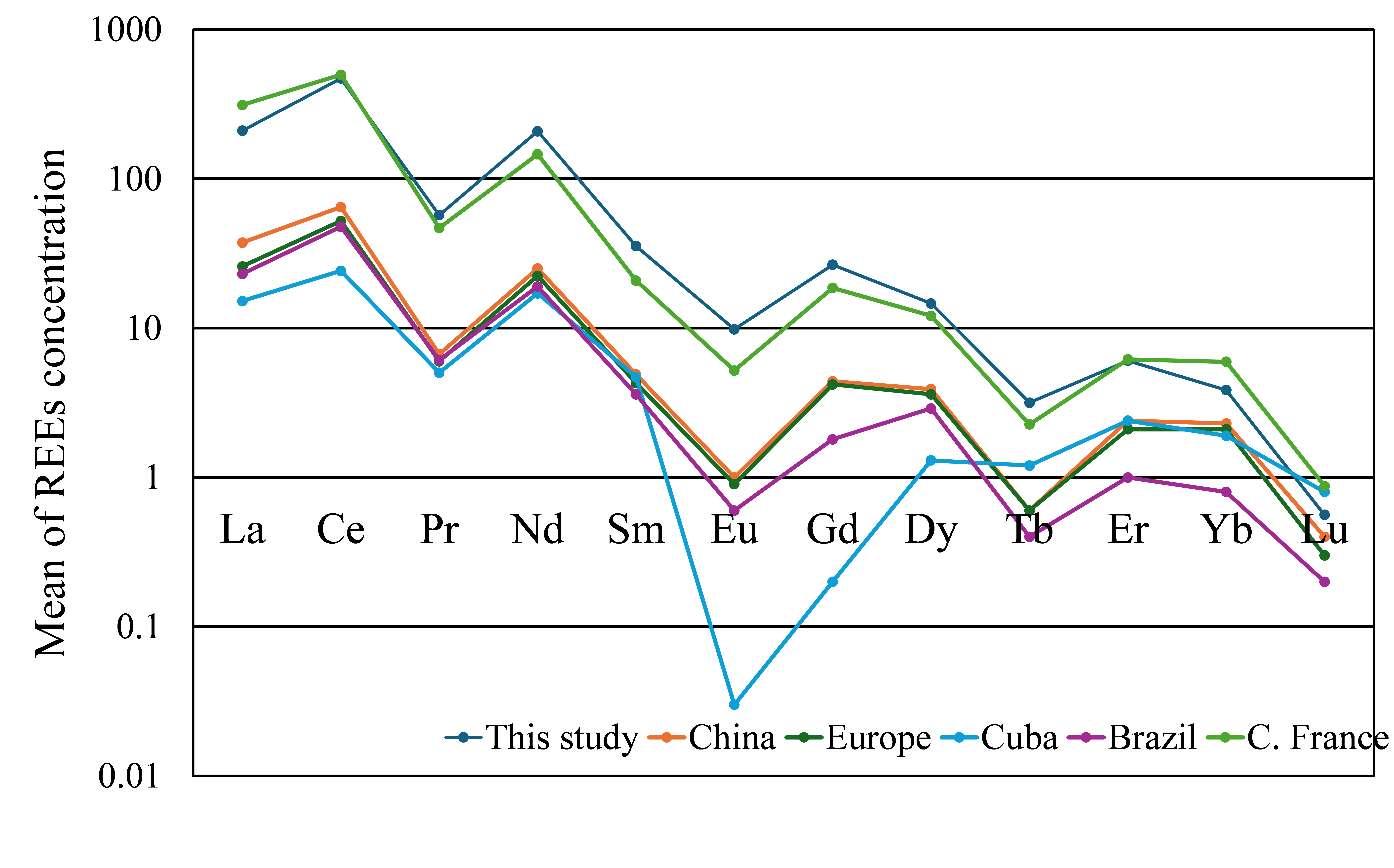


**Fig. S1** Mean of REEs concentration of the soil samples in this study, with literature information. The values of REEs concentration in soil samples were obtained from Alfaro et al. 2018; Brioschi et al. 2013; Landim et al. 2022; Wei et al. 1991


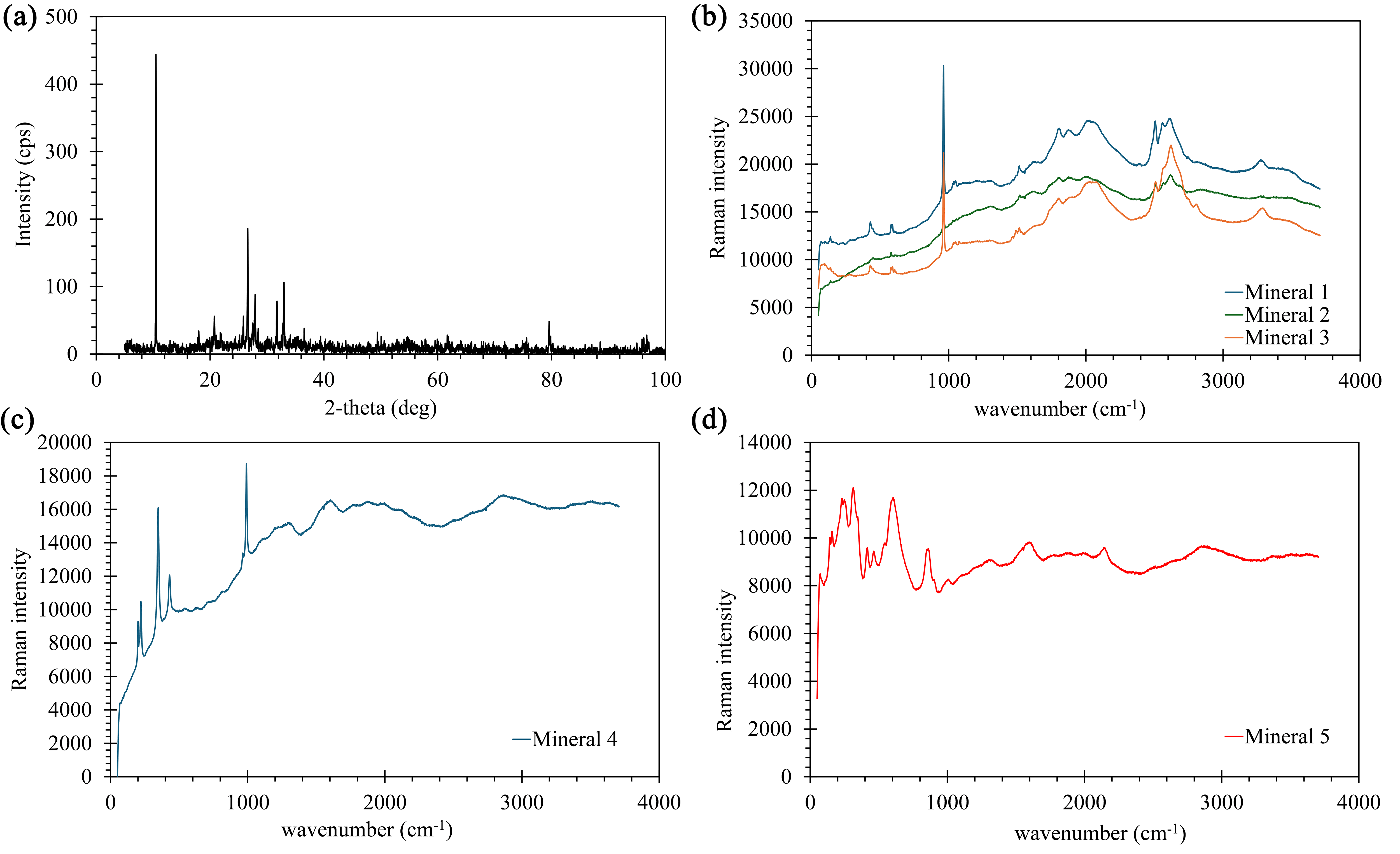


**Fig. S2** Mineralogical analysis of PFC017S sample. XRD of total sample (a), Raman analysis of minerals identified (b, c and d)


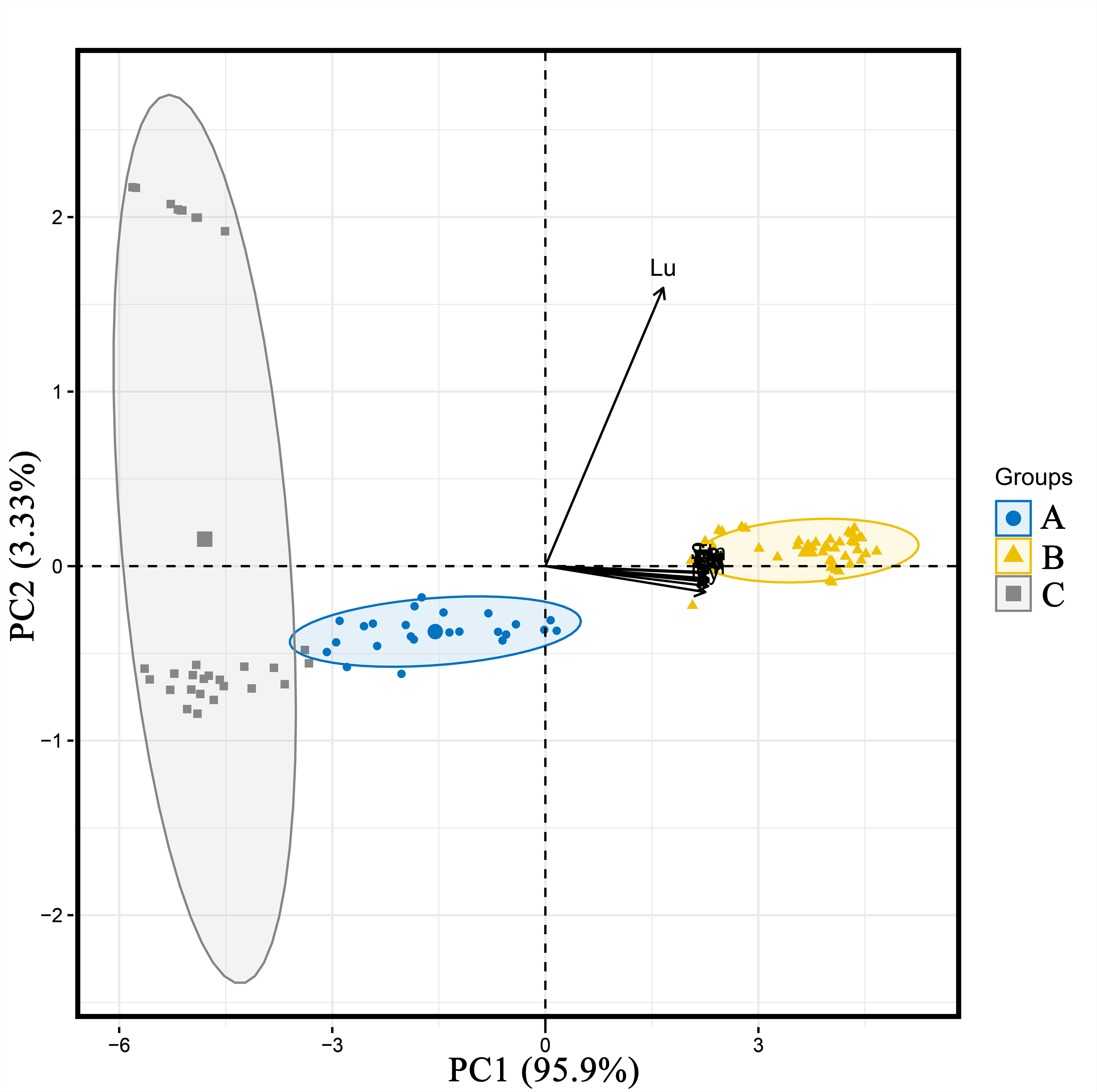


**Fig. S3** PCA graph correlating samples of rocks, soil, roots, and leaves

A thorough examination of the diffractogram of sample PFC017S (Figure S2a) reveals the presence of peaks indicative of a quartz and clay matrix, in addition to the consistent indication of phosphate/carbonate phases, which are enriched with REEs. Specifically, the peak at 26.59° (d = 3.349 Å) exhibits a strong correspondence to quartz, while the signal at 10.49° (d = 8.424 Å) is consistent with the 00^1^ reflection of clay minerals (illite/interstratified) (Saminpanya and Denkitkul 2020). Furthermore, the adjacent peaks at 31.74° (d = 2.817 Å) and 32.918° (d = 2.7187 Å) are indicative of rare earth phosphates/carbonates, such as apatite, monazite, xenotime, or bastnäsite (Bamforth et al. 2024). These interpretations were corroborated by Raman spectroscopy, which allowed for direct identification of the phosphate and complex oxide phases. As illustrated in Figure S2b, the peaks at 962, 432, 580, and 589 cm^‒1^ were identified as being attributed to fluoroapatite, corresponding respectively to the ν1 (PO_4_^3-^) mode, the ν2 (PO_4_^3-)^ bending mode, and the ν4 (PO_4_^3-^) bending mode (Penel et al. 1997). As illustrated in Figure S2c, the spectrum exhibited an intense band at 990 cm-1, accompanied by signals at 430 and 347 cm^‒1^ and a doublet at 201 and 222 cm^‒1^. These characteristics are indicative of monazite - (Ce, La, Nd) (Clavier et al. 2018). As illustrated in Figure S2d, the presence of multiple doublets between 140–250 cm^‒1^ and peaks at 315.5, 462, 543, and 604 cm^‒1^ signifies the occurrence of complex oxides from the aeschynite–polycrase group, which is abundant in Nb, Ti, and REEs. The vibrational signature of these oxides reflects lattice modes involving heavy cations and Nb–O/Ti–O stretches in distorted octahedra (Guastoni et al. 2019; Nasir et al. 2009). Therefore, the correlation between XRD and Raman spectroscopy indicates the coexistence of a predominantly silicate/clay matrix with accessory phases of geochemical interest, including apatite, monazite, and complex oxides (aeschynite/polycrase). These minerals are consistent with the carbonatitic nature of the studied area (Cerva-Alves et al. 2017; Morales et al. 2019). These findings, in conjunction with the elevated concentrations of REEs as determined by ICP-MS, suggest the possibility that a proportion of the REEs present in the soil sample are effectively immobilized within phosphate minerals. Concurrently, a separate fraction may be associated with clays and oxides, thereby functioning as adsorbent phases. This mineralogical distribution helps explain the mobility and differential retention of REEs in the carbonatite-soil system.

**Table S1** Concentration of REEs (mg kg^‒1^) in soil samples

| **Sample** | **La** | **Ce** | **Pr** | **Nd** | **Sm** | **Eu** | **Gd** | **Dy** | **Tb** | **Ho** | **Er** | **Tm** | **Yb** | **Lu** | **Y** | **ΣREEs** | **ΣLREEs** | **ΣHREEs** | **ΣLREEs /ΣHREEs** |
| --- | --- | --- | --- | --- | --- | --- | --- | --- | --- | --- | --- | --- | --- | --- | --- | --- | --- | --- | --- |
| PFC001S | 63.8 | 136 | 16.7 | 60.7 | 12.6 | 3.49 | 10 | 7.6 | 1.5 | 1.3 | 3.6 | 0.5 | 3 | 0.5 | 33.3 | 354.6 | 293.3 | 61.3 | 4.8 |
| PFC002S | 356 | 793 | 100 | 355 | 57.6 | 16.6 | 44.3 | 23.9 | 5.2 | 3.9 | 9.3 | 1.2 | 5.8 | 0.8 | 87.9 | 1860.5 | 1678.2 | 182.3 | 9.2 |
| PFC003S | 239 | 562 | 69.4 | 252 | 44 | 12.4 | 33.6 | 16.9 | 3.8 | 2.7 | 6 | 0.8 | 3.6 | 0.5 | 57.3 | 1304.0 | 1178.8 | 125.2 | 9.4 |
| PFC004S | 202 | 462 | 59.6 | 226 | 41.1 | 11.7 | 31.2 | 18.1 | 3.8 | 2.9 | 7.6 | 1 | 4.9 | 0.7 | 70.8 | 1143.4 | 1002.4 | 141 | 7.1 |
| PFC005S | 283 | 679 | 88.1 | 321 | 52 | 13.4 | 34.1 | 16.1 | 3.8 | 2.3 | 5.4 | 0.6 | 2.6 | 0.4 | 49.1 | 1550.9 | 1436.5 | 114.4 | 12.6 |
| PFC006S | 245 | 578 | 73.5 | 274 | 44.2 | 12.2 | 32.3 | 17.6 | 3.8 | 2.9 | 7.2 | 0.8 | 3.7 | 0.5 | 68 | 1363.7 | 1226.9 | 136.8 | 9.0 |
| PFC007S | 283 | 630 | 82.4 | 306 | 49.3 | 12.9 | 34 | 16.5 | 3.7 | 2.5 | 5.8 | 0.7 | 3.1 | 0.4 | 56 | 1486.3 | 1363.6 | 122.7 | 11.1 |
| PFC008S | 318 | 726 | 92.7 | 339 | 56.6 | 15.2 | 41.4 | 20.3 | 4.7 | 3.2 | 7.4 | 0.8 | 4.1 | 0.6 | 68.2 | 1698.2 | 1547.5 | 150.7 | 10.3 |
| PFC009S | 259 | 602 | 77.2 | 290 | 49.2 | 13.6 | 36.4 | 18.4 | 4.2 | 2.9 | 6.9 | 0.8 | 3.8 | 0.5 | 63.8 | 1428.7 | 1291.0 | 137.7 | 9.4 |
| PFC010S | 204 | 472 | 59.6 | 222 | 38.5 | 11.2 | 30.5 | 19.1 | 4 | 3.4 | 8.9 | 1.1 | 5.7 | 0.8 | 79 | 1159.8 | 1007.3 | 152.5 | 6.6 |
| PFC011S | 72.5 | 157 | 17.9 | 63.6 | 11.8 | 2.36 | 8.6 | 4.8 | 1.1 | 0.7 | 1.9 | 0.2 | 1.6 | 0.2 | 17.7 | 362.0 | 325.2 | 36.8 | 8.8 |
| PFC012S | 70.3 | 164 | 17 | 63.8 | 11.6 | 2.14 | 7.9 | 3.6 | 0.9 | 0.5 | 1.2 | 0.2 | 0.9 | 0.1 | 11.1 | 355.2 | 328.8 | 26.4 | 12.5 |
| PFC013S | 74.2 | 172 | 18.4 | 68 | 12 | 2.15 | 8.6 | 4.1 | 0.9 | 0.6 | 1.6 | 0.2 | 1.2 | 0.2 | 14.3 | 378.5 | 346.8 | 31.7 | 10.9 |
| PFC014S | 74 | 180 | 18.2 | 67.6 | 11.9 | 2.27 | 8.9 | 4.1 | 1 | 0.7 | 1.7 | 0.3 | 1.4 | 0.2 | 16.6 | 388.9 | 354.0 | 34.9 | 10.1 |
| PFC015S | 54.9 | 127 | 14.3 | 54.3 | 9.4 | 1.76 | 8.2 | 4.4 | 1 | 0.7 | 1.7 | 0.3 | 1.5 | 0.3 | 16 | 295.8 | 261.7 | 34.1 | 7.7 |
| PFC016S | 44 | 103 | 11.8 | 44.9 | 9 | 2.46 | 7.1 | 5 | 1 | 0.9 | 2.6 | 0.4 | 2.1 | 0.3 | 23.7 | 258.3 | 215.2 | 43.1 | 5.0 |
| PFC017S | 426 | 984 | 117 | 418 | 62 | 19 | 49.9 | 26.4 | 5.9 | 4.2 | 10.3 | 1.2 | 6 | 0.9 | 101 | 2231.8 | 2026.0 | 205.8 | 9.8 |
| PFC018S | 332 | 771 | 92.1 | 337 | 58.2 | 16 | 43.6 | 23.5 | 5.1 | 3.7 | 9 | 1.1 | 5.3 | 0.7 | 83.5 | 1781.8 | 1606.3 | 175.5 | 9.2 |
| PFC019S | 39.9 | 103 | 12.1 | 45.4 | 9.3 | 1.82 | 7.1 | 3.8 | 0.9 | 0.7 | 1.7 | 0.2 | 1.6 | 0.2 | 12.4 | 240.1 | 211.5 | 28.6 | 7.4 |
| PTC001S | 328 | 731 | 86.5 | 310 | 52.7 | 14.9 | 39.7 | 21.3 | 4.5 | 3.5 | 8.7 | 1.1 | 5.9 | 0.8 | 83.8 | 1692.4 | 1523.1 | 169.3 | 9.0 |
| PTC002S | 103 | 209 | 23.8 | 87.6 | 17.6 | 4.45 | 12.3 | 7.5 | 1.6 | 1.3 | 3.6 | 0.5 | 2.6 | 0.4 | 32.9 | 508.2 | 445.5 | 62.7 | 7.1 |
| PTC003S | 50.4 | 95.3 | 12.4 | 45.9 | 9.2 | 2.18 | 7.3 | 5.5 | 1 | 1 | 3 | 0.4 | 2.5 | 0.4 | 26.9 | 263.4 | 215.4 | 48 | 4.5 |
| PTC004S | 202 | 411 | 46.5 | 166 | 30.2 | 8.04 | 21.9 | 12.6 | 2.6 | 2.1 | 5.5 | 0.7 | 3.8 | 0.6 | 51.9 | 965.4 | 863.7 | 101.7 | 8.5 |
| PTC005S | 211 | 425 | 47.6 | 172 | 31.3 | 8.54 | 24 | 12.9 | 2.8 | 2.2 | 5.8 | 0.7 | 3.8 | 0.6 | 56.4 | 1004.6 | 895.4 | 109.2 | 8.2 |
| PTC006S | 140 | 268 | 28.5 | 105 | 20.9 | 6.16 | 16.9 | 9.8 | 2.1 | 1.6 | 4.1 | 0.5 | 2.9 | 0.4 | 39.9 | 646.8 | 568.6 | 78.2 | 7.3 |
| PTC007S | 238 | 561 | 59.4 | 208 | 35.2 | 9.79 | 25.8 | 14.1 | 3 | 2.4 | 6.2 | 0.8 | 4 | 0.6 | 58.3 | 1226.6 | 1111.4 | 115.2 | 9.6 |
| PTC008S | 65.5 | 116 | 15.6 | 58 | 11.2 | 2.85 | 9.5 | 7.1 | 1.4 | 1.3 | 3.9 | 0.5 | 3.1 | 0.5 | 34.2 | 330.7 | 269.2 | 61.5 | 4.4 |
| PTC009S | 64.3 | 136 | 14.1 | 49.9 | 9.6 | 2.2 | 7.3 | 5.1 | 1 | 0.9 | 2.9 | 0.4 | 2.5 | 0.4 | 24.2 | 320.8 | 276.1 | 44.7 | 6.2 |
| PTC010S | 177 | 376 | 40.8 | 145 | 24.3 | 6.73 | 18.5 | 11.2 | 2.3 | 1.9 | 5.4 | 0.7 | 3.7 | 0.6 | 47 | 861.1 | 769.8 | 91.3 | 8.4 |

**Table S2** Groups and components of PCA of Figure 2a

| **A** | **B** | **C** | **D** |
| --- | --- | --- | --- |
| PFC003S | PFC011S | PFC001S | PFC002S |
| PFC004S | PFC012S | PTC002S | PFC008S |
| PFC005S | PFC013S | PTC006S | PFC017S |
| PFC006S | PFC014S | PTC008S | PFC019S |
| PFC007S | PFC015S | PTC010S | PTC001S |
| PFC009S | PFC016S |  |  |
| PTC004S | PFC019S |  |  |
| PTC005S | PTC003S |  |  |
| PTC007S | PTC009S |  |  |

**Table S3** Concentration of major elements (%), ΣREEs (mg kg^‒1^), and pH in soil samples

| **Sample** | **P_2_O_5_** | **Na_2_O** | **MgO** | **K_2_O** | **Fe_2_O_3_** | **Al_2_O_3_** | **TiO_2_** | **CaO** | **pH** | **ΣREEs** | **Al_2_O_3_/CaO** |
| --- | --- | --- | --- | --- | --- | --- | --- | --- | --- | --- | --- |
| PFC001S | 0.277 | 2.6325 | 2.706 | 1.104 | 13.73 | 13.42 | 0.184 | 2.84 | 5.1 | 354.6 | 4.72 |
| PFC002S | 0.289 | 1.0935 | 3.287 | 0.624 | 17.16 | 10.17 | 0.301 | 10.37 | 5.6 | 1860.5 | 0.980 |
| PFC003S | 0.350 | 0.972 | 4.133 | 0.54 | 16.87 | 9.49 | 0.301 | 12.67 | 5.6 | 1304.0 | 0.749 |
| PFC004S | 0.435 | 1.134 | 2.473 | 1.104 | 15.30 | 11.55 | 0.150 | 10.32 | 5.4 | 1143.4 | 1.119 |
| PFC005S | 1.021 | 0.5535 | 4.764 | 0.312 | 17.30 | 9.03 | 0.367 | 10.84 | 5.4 | 1550.9 | 0.834 |
| PFC006S | 1.429 | 0.8505 | 3.154 | 0.516 | 19.31 | 8.60 | 0.451 | 10.18 | 5.4 | 1363.7 | 0.845 |
| PFC007S | 0.733 | 0.5805 | 3.353 | 0.6 | 17.16 | 8.79 | 0.317 | 10.49 | 5.5 | 1486.3 | 0.838 |
| PFC008S | 0.463 | 0.81 | 3.901 | 0.624 | 16.59 | 9.07 | 0.317 | 13.22 | 5.5 | 1698.2 | 0.686 |
| PFC009S | 0.554 | 0.864 | 3.021 | 0.36 | 16.59 | 7.75 | 0.251 | 12.56 | 5.4 | 1428.7 | 0.617 |
| PFC010S | 0.387 | 1.0665 | 3.287 | 0.9 | 17.02 | 10.47 | 0.284 | 10.98 | 5.4 | 1159.8 | 0.954 |
| PFC011S | 0.149 | 0.2565 | 1.046 | 1.164 | 9.61 | 15.04 | 0.251 | 0.31 | 4.5 | 362.0 | 48.8 |
| PFC012S | 0.183 | 0.405 | 1.245 | 1.764 | 10.60 | 16.88 | 0.217 | 0.49 | 4.2 | 355.2 | 34.5 |
| PFC013S | 0.167 | 0.3105 | 0.913 | 1.632 | 8.61 | 15.91 | 0.284 | 0.48 | 4.4 | 378.5 | 33.4 |
| PFC014S | 0.151 | 0.2565 | 0.813 | 1.38 | 9.51 | 16.16 | 0.317 | 0.28 | 4.5 | 388.9 | 57.7 |
| PFC015S | 0.080 | 0.297 | 1.079 | 1.068 | 6.86 | 11.51 | 0.200 | 0.31 | 4.5 | 295.8 | 37.3 |
| PFC016S | 0.286 | 1.728 | 2.639 | 1.92 | 12.37 | 13.59 | 0.317 | 3.40 | 5.2 | 258.3 | 3.99 |
| PFC017S | 1.367 | 1.4715 | 3.088 | 0.552 | 15.02 | 9.15 | 0.050 | 11.07 | 5.3 | 2231.8 | 0.826 |
| PFC018S | 0.263 | 0.9315 | 2.789 | 0.576 | 16.30 | 8.01 | 0.284 | 13.48 | 5.4 | 1781.8 | 0.594 |
| PFC019S | 0.202 | 0.2025 | 0.730 | 1.2 | 9.34 | 13.15 | 0.752 | 0.31 | 5.4 | 240.1 | 42.7 |
| PTC001S | 0.953 | 1.6065 | 2.49 | 1.128 | 10.90 | 11.15 | 0.050 | 6.73 | 7.1 | 1692.4 | 1.65 |
| PTC002S | 0.238 | 2.106 | 1.195 | 1.116 | 10.18 | 11.89 | 0.167 | 1.72 | 6.7 | 508.2 | 6.90 |
| PTC003S | 0.137 | 1.755 | 2.556 | 1.608 | 6.99 | 12.32 | 0.100 | 2.62 | 6.5 | 263.4 | 4.70 |
| PTC004S | 0.417 | 3.1185 | 1.776 | 0.876 | 11.35 | 12.87 | 0.301 | 3.07 | 6.2 | 965.4 | 4.19 |
| PTC005S | 0.520 | 3.6045 | 2.507 | 0.78 | 13.64 | 13.99 | 0.351 | 3.43 | 6 | 1004.6 | 4.07 |
| PTC006S | 0.419 | 3.267 | 3.901 | 1.536 | 14.19 | 13.31 | 0.167 | 4.70 | 5.9 | 646.8 | 2.82 |
| PTC007S | 0.144 | 1.7685 | 1.328 | 0.864 | 11.61 | 12.38 | 0.150 | 3.67 | 5.8 | 1226.6 | 3.37 |
| PTC008S | 0.153 | 1.5255 | 1.992 | 1.644 | 6.51 | 13.44 | 0.150 | 2.14 | 5.6 | 330.7 | 6.27 |
| PTC009S | 0.089 | 1.8495 | 0.681 | 1.764 | 4.36 | 12.95 | 0.267 | 1.06 | 5.5 | 320.8 | 12.2 |
| PTC010S | 0.229 | 1.9845 | 1.328 | 1.44 | 10.00 | 13.12 | 0.150 | 1.78 | 5.6 | 861.1 | 7.38 |

**Table S4** Concentration of REEs (mg kg^‒1^) in vegetation samples

| **Sample** | **La** | **Ce** | **Pr** | **Nd** | **Sm** | **Eu** | **Gd** | **Dy** | **Tb** | **Ho** | **Er** | **Tm** | **Yb** | **Lu** | **Y** | **ΣREEs** | **ΣLREEs** | **ΣHREEs** | **ΣLREEs/**  **ΣHREEs** |
| --- | --- | --- | --- | --- | --- | --- | --- | --- | --- | --- | --- | --- | --- | --- | --- | --- | --- | --- | --- |
| PFC001R | 1.9 | 2.73 | 0.349 | 1.37 | 0.212 | 0.0567 | 0.186 | 0.134 | 0.0226 | 0.0264 | 0.0702 | 0.0093 | 0.0537 | 0.0075 | 0.683 | 7.8104 | 6.6177 | 1.1927 | 0.00555 |
| PFC002R | 10.9 | 19.2 | 2.38 | 8.84 | 1.32 | 0.321 | 0.964 | 0.583 | 0.101 | 0.0902 | 0.236 | 0.0288 | 0.166 | 0.0208 | 2.54 | 47.6908 | 42.961 | 4.7298 | 0.00908 |
| PFC003R | 19.9 | 36.1 | 4.27 | 16.5 | 2.34 | 0.586 | 1.61 | 0.862 | 0.163 | 0.134 | 0.312 | 0.0348 | 0.193 | 0.0251 | 3.31 | 86.3399 | 79.696 | 6.6439 | 0.012 |
| PFC004R | 15.5 | 29.6 | 3.52 | 13.9 | 2.03 | 0.505 | 1.43 | 0.8 | 0.148 | 0.133 | 0.318 | 0.0377 | 0.238 | 0.0284 | 3.43 | 71.6181 | 65.055 | 6.5631 | 0.00991 |
| PFC005R | 11.9 | 19.8 | 2.51 | 9.82 | 1.34 | 0.329 | 0.934 | 0.486 | 0.0916 | 0.079 | 0.174 | 0.02 | 0.125 | 0.0163 | 1.99 | 49.6149 | 45.699 | 3.9159 | 0.01167 |
| PFC006R | 9.93 | 16 | 2.05 | 7.96 | 1.09 | 0.272 | 0.812 | 0.476 | 0.0818 | 0.0773 | 0.192 | 0.021 | 0.127 | 0.0162 | 2.1 | 41.2053 | 37.302 | 3.9033 | 0.00956 |
| PFC007R | 4.34 | 7.11 | 0.949 | 3.68 | 0.507 | 0.119 | 0.322 | 0.164 | 0.0318 | 0.0238 | 0.054 | 0.0062 | 0.0298 | 0.0041 | 0.617 | 17.9577 | 16.705 | 1.2527 | 0.01334 |
| PFC008R | 10.8 | 19.7 | 2.36 | 9.3 | 1.3 | 0.321 | 0.931 | 0.474 | 0.0924 | 0.0746 | 0.17 | 0.0204 | 0.118 | 0.0145 | 1.88 | 47.5559 | 43.781 | 3.7749 | 0.0116 |
| PFC009R | 18.3 | 30.6 | 3.61 | 13.8 | 1.9 | 0.474 | 1.38 | 0.743 | 0.139 | 0.117 | 0.285 | 0.0319 | 0.187 | 0.0233 | 3.05 | 74.6402 | 68.684 | 5.9562 | 0.01153 |
| PFC010R | 8.64 | 13.5 | 1.63 | 6.18 | 0.848 | 0.222 | 0.679 | 0.421 | 0.0716 | 0.0724 | 0.19 | 0.0221 | 0.132 | 0.0202 | 2.01 | 34.6383 | 31.02 | 3.6183 | 0.00857 |
| PFC011R | 1.98 | 3.53 | 0.435 | 1.73 | 0.342 | 0.0869 | 0.316 | 0.255 | 0.0403 | 0.0417 | 0.122 | 0.0153 | 0.102 | 0.0134 | 1.15 | 10.1596 | 8.1039 | 2.0557 | 0.00394 |
| PFC012R | 0.75 | 1.38 | 0.155 | 0.593 | 0.101 | 0.0214 | 0.0721 | 0.0442 | 0.0087 | 0.0069 | 0.0204 | 0.0026 | 0.0143 | 0.0018 | 0.202 | 3.3734 | 3.0004 | 0.373 | 0.00804 |
| PFC013R | 2.33 | 4.58 | 0.495 | 1.93 | 0.36 | 0.0737 | 0.293 | 0.208 | 0.0361 | 0.0335 | 0.0921 | 0.0121 | 0.077 | 0.0093 | 0.926 | 11.4558 | 9.7687 | 1.6871 | 0.00579 |
| PFC014R | 0.98 | 1.82 | 0.194 | 0.785 | 0.129 | 0.0366 | 0.137 | 0.103 | 0.0169 | 0.0178 | 0.0546 | 0.0077 | 0.0493 | 0.0062 | 0.583 | 4.9201 | 3.9446 | 0.9755 | 0.00404 |
| PFC015R | 2.33 | 4.08 | 0.478 | 1.91 | 0.339 | 0.0973 | 0.316 | 0.247 | 0.0383 | 0.0462 | 0.131 | 0.0172 | 0.115 | 0.0162 | 1.19 | 11.3512 | 9.2343 | 2.1169 | 0.00436 |
| PTC001R | 4.57 | 9.2 | 1.12 | 4.59 | 0.714 | 0.186 | 0.556 | 0.333 | 0.0607 | 0.0559 | 0.137 | 0.0169 | 0.0979 | 0.0123 | 1.44 | 23.0897 | 20.38 | 2.7097 | 0.00752 |
| PTC002R | 1.27 | 1.52 | 0.229 | 0.899 | 0.145 | 0.0356 | 0.112 | 0.0648 | 0.0112 | 0.0102 | 0.0263 | 0.0035 | 0.0215 | 0.0025 | 0.327 | 4.6776 | 4.0986 | 0.579 | 0.00708 |
| PTC003R | 3.81 | 5.83 | 0.762 | 3.09 | 0.515 | 0.116 | 0.423 | 0.292 | 0.0479 | 0.0508 | 0.145 | 0.0181 | 0.112 | 0.0149 | 1.45 | 16.6767 | 14.123 | 2.5537 | 0.00553 |
| PTC004R | 2.38 | 3.83 | 0.466 | 1.8 | 0.28 | 0.0719 | 0.214 | 0.139 | 0.0235 | 0.0218 | 0.057 | 0.0074 | 0.0445 | 0.0054 | 0.615 | 9.9555 | 8.8279 | 1.1276 | 0.00783 |
| PTC005R | 3.68 | 5.53 | 0.668 | 2.64 | 0.393 | 0.101 | 0.322 | 0.19 | 0.0334 | 0.0319 | 0.0832 | 0.0098 | 0.0587 | 0.008 | 0.889 | 14.638 | 13.012 | 1.626 | 0.008 |
| PTC006R | 2.78 | 3.62 | 0.363 | 1.26 | 0.163 | 0.0392 | 0.122 | 0.0799 | 0.0126 | 0.0133 | 0.0328 | 0.0036 | 0.026 | 0.0042 | 0.351 | 8.8706 | 8.2252 | 0.6454 | 0.01274 |
| PTC007R | 4.64 | 9.19 | 1.02 | 4.04 | 0.626 | 0.161 | 0.488 | 0.296 | 0.0505 | 0.0479 | 0.12 | 0.015 | 0.0914 | 0.0113 | 1.28 | 22.0771 | 19.677 | 2.4001 | 0.0082 |
| PTC008R | 1.65 | 2.45 | 0.329 | 1.27 | 0.224 | 0.0467 | 0.174 | 0.126 | 0.0223 | 0.0227 | 0.0626 | 0.0074 | 0.0489 | 0.0067 | 0.643 | 7.0833 | 5.9697 | 1.1136 | 0.00536 |
| PTC009R | 1.14 | 1.79 | 0.2 | 0.749 | 0.118 | 0.0263 | 0.098 | 0.0653 | 0.0113 | 0.0109 | 0.0261 | 0.0037 | 0.0227 | 0.003 | 0.307 | 4.5713 | 4.0233 | 0.548 | 0.00734 |
| PTC010R | 3.3 | 5.53 | 0.67 | 2.7 | 0.415 | 0.112 | 0.346 | 0.201 | 0.0371 | 0.0328 | 0.0851 | 0.0103 | 0.06 | 0.0078 | 0.956 | 14.4631 | 12.727 | 1.7361 | 0.00733 |
| PFC001P | 0.46 | 0.721 | 0.086 | 0.342 | 0.062 | 0.0136 | 0.0484 | 0.0375 | 0.0061 | 0.0065 | 0.0179 | 0.0022 | 0.0156 | 0.0015 | 0.176 | 1.9963 | 1.6846 | 0.3117 | 0.0054 |
| PFC002P | 0.5 | 0.629 | 0.094 | 0.365 | 0.056 | 0.0136 | 0.038 | 0.0218 | 0.0042 | 0.0041 | 0.008 | 0.0011 | 0.007 | 0.0007 | 0.135 | 1.8775 | 1.6576 | 0.2199 | 0.00754 |
| PFC003P | 0.53 | 0.33 | 0.083 | 0.312 | 0.04 | 0.0105 | 0.0336 | 0.0149 | 0.0029 | 0.0029 | 0.0072 | 0.0007 | 0.0037 | <LD | 0.128 | 1.4994 | 1.3055 | 0.1939 | 0.00673 |
| PFC004P | 0.35 | 0.43 | 0.063 | 0.217 | 0.039 | 0.0082 | 0.0216 | 0.0121 | 0.0022 | 0.0021 | 0.005 | 0.0005 | 0.0024 | <LD | 0.059 | 1.2121 | 1.1072 | 0.1049 | 0.01055 |
| PFC005P | 0.5 | 0.719 | 0.094 | 0.374 | 0.049 | 0.0112 | 0.0369 | 0.0195 | 0.0036 | 0.0031 | 0.006 | 0.0008 | 0.0045 | 0.0005 | 0.083 | 1.9051 | 1.7472 | 0.1579 | 0.01107 |
| PFC006P | 0.43 | 0.527 | 0.082 | 0.316 | 0.043 | 0.0102 | 0.029 | 0.0172 | 0.0027 | 0.0028 | 0.0052 | 0.0007 | 0.005 | 0.0005 | 0.107 | 1.5783 | 1.4082 | 0.1701 | 0.00828 |
| PFC007P | 0.32 | 0.319 | 0.059 | 0.222 | 0.029 | 0.006 | 0.0178 | 0.01 | 0.0016 | 0.0017 | 0.0039 | 0.0004 | 0.0027 | <LD | 0.051 | 1.0441 | 0.955 | 0.0891 | 0.01072 |
| PFC008P | 0.75 | 1.25 | 0.164 | 0.642 | 0.087 | 0.0223 | 0.0623 | 0.0331 | 0.006 | 0.005 | 0.013 | 0.0015 | 0.0082 | 0.0011 | 0.143 | 3.1885 | 2.9153 | 0.2732 | 0.01067 |
| PFC009P | 2.11 | 3.78 | 0.428 | 1.66 | 0.223 | 0.0537 | 0.159 | 0.0944 | 0.0162 | 0.0145 | 0.0351 | 0.0046 | 0.0242 | 0.0031 | 0.393 | 8.9988 | 8.2547 | 0.7441 | 0.01109 |
| PFC010P | 1.57 | 2.46 | 0.293 | 1.15 | 0.151 | 0.041 | 0.127 | 0.0773 | 0.0124 | 0.0134 | 0.0324 | 0.0041 | 0.0226 | 0.0034 | 0.364 | 6.3216 | 5.665 | 0.6566 | 0.00863 |
| PFC011P | 0.42 | 0.542 | 0.077 | 0.28 | 0.301 | 0.0111 | 0.0369 | 0.0224 | 0.0038 | 0.0048 | 0.011 | 0.0014 | 0.0053 | 0.0009 | 0.183 | 1.9006 | 1.6311 | 0.2695 | 0.00605 |
| PFC012P | 0.29 | 0.469 | 0.047 | 0.196 | 0.031 | 0.0071 | 0.025 | 0.0151 | 0.0023 | 0.0025 | 0.0063 | 0.001 | 0.0047 | 0.0008 | 0.098 | 1.1958 | 1.0401 | 0.1557 | 0.00668 |
| PFC013P | 0.3 | 0.307 | 0.049 | 0.181 | 0.027 | 0.007 | 0.0317 | 0.0228 | 0.0038 | 0.0038 | 0.011 | 0.0017 | 0.0065 | 0.0011 | 0.208 | 1.1614 | 0.871 | 0.2904 | 0.003 |
| PFC014P | 0.32 | 0.363 | 0.055 | 0.21 | 0.032 | 0.0087 | 0.0283 | 0.0194 | 0.0032 | 0.0037 | 0.0098 | 0.0011 | 0.0069 | 0.0009 | 0.157 | 1.219 | 0.9887 | 0.2303 | 0.00429 |
| PFC015P | 0.21 | 0.246 | 0.033 | 0.13 | 0.018 | 0.0049 | 0.0156 | 0.0118 | 0.0018 | 0.0019 | 0.0056 | 0.0007 | 0.0039 | 0.0007 | 0.094 | 0.7779 | 0.6419 | 0.136 | 0.00472 |
| PFC016P | 0.43 | 0.264 | 0.058 | 0.216 | 0.025 | 0.0064 | 0.0239 | 0.0143 | 0.0026 | 0.0025 | 0.0063 | 0.0006 | 0.0055 | 0.0006 | 0.106 | 1.1617 | 0.9994 | 0.1623 | 0.00616 |
| PFC017P | 0.4 | 0.464 | 0.07 | 0.272 | 0.04 | 0.0092 | 0.0308 | 0.0163 | 0.0031 | 0.0031 | 0.0075 | 0.001 | 0.0063 | 0.0007 | 0.096 | 1.42 | 1.2552 | 0.1648 | 0.00762 |
| PFC018P | 0.26 | 0.283 | 0.046 | 0.183 | 0.027 | 0.0049 | 0.0248 | 0.0102 | 0.0017 | 0.0016 | 0.004 | 0.0005 | 0.003 | <LD | 0.056 | 0.9057 | 0.8039 | 0.1018 | 0.0079 |
| PFC019P | 0.34 | 0.412 | 0.069 | 0.273 | 0.04 | 0.0113 | 0.0398 | 0.0248 | 0.0043 | 0.0039 | 0.0104 | 0.0012 | 0.0076 | 0.001 | 0.174 | 1.4123 | 1.1453 | 0.267 | 0.00429 |
| PTC001P | 0.18 | 0.242 | 0.041 | 0.173 | 0.03 | 0.0068 | 0.0241 | 0.0113 | 0.0023 | 0.0026 | 0.0064 | 0.0007 | 0.0044 | <LD | 0.097 | 0.8216 | 0.6728 | 0.1488 | 0.00452 |
| PTC002P | 0.4 | 0.455 | 0.081 | 0.307 | 0.064 | 0.0092 | 0.0327 | 0.0202 | 0.0037 | 0.0034 | 0.011 | 0.0011 | 0.0049 | 0.0009 | 0.126 | 1.5201 | 1.3162 | 0.2039 | 0.00646 |
| PTC003P | 0.16 | 0.279 | 0.036 | 0.135 | 0.025 | 0.0062 | 0.0127 | 0.0108 | 0.0017 | 0.0021 | 0.0046 | 0.0006 | 0.0044 | <LD | 0.06 | 0.7381 | 0.6412 | 0.0969 | 0.00662 |
| PTC004P | 0.17 | 0.192 | 0.025 | 0.092 | 0.016 | 0.0026 | 0.0102 | 0.0057 | 0.0009 | 0.0011 | 0.0028 | 0.0003 | 0.0016 | <LD | 0.039 | 0.5592 | 0.4976 | 0.0616 | 0.00808 |
| PTC005P | 0.49 | 0.377 | 0.075 | 0.305 | 0.047 | 0.0124 | 0.0392 | 0.0203 | 0.0035 | 0.0034 | 0.0073 | 0.001 | 0.0042 | 0.0007 | 0.156 | 1.542 | 1.3064 | 0.2356 | 0.00554 |
| PTC006P | 0.19 | 0.26 | 0.034 | 0.125 | 0.052 | 0.0049 | 0.0124 | 0.0096 | 0.0015 | 0.0017 | 0.0036 | 0.0004 | 0.0038 | <LD | 0.044 | 0.7429 | 0.6659 | 0.077 | 0.00865 |
| PTC007P | 0.45 | 0.624 | 0.098 | 0.417 | 0.059 | 0.0142 | 0.0467 | 0.0256 | 0.0046 | 0.0042 | 0.01 | 0.0011 | 0.0057 | 0.001 | 0.137 | 1.8981 | 0.0989 | 0.2359 | 0.00042 |
| PTC008P | 0.13 | 0.188 | 0.023 | 0.089 | 0.03 | 0.0027 | 0.0101 | 0.0082 | 0.0012 | 0.0011 | 0.0022 | 0.0002 | 0.0021 | <LD | 0.04 | 0.5278 | 0.0251 | 0.0651 | 0.00039 |
| PTC009P | 0.33 | 0.308 | 0.046 | 0.195 | 0.029 | 0.0051 | 0.0203 | 0.0107 | 0.0019 | 0.0017 | 0.0044 | 0.0006 | 0.0024 | 0.0006 | 0.076 | 1.0317 | 0.0426 | 0.1186 | 0.00036 |
| PTC010P | 1.05 | 0.741 | 0.231 | 0.957 | 0.123 | 0.0323 | 0.106 | 0.046 | 0.0091 | 0.0081 | 0.0191 | 0.0022 | 0.0104 | 0.0015 | 0.337 | 3.6737 | 0.2024 | 0.5394 | 0.00038 |

**Table S5** BAF, calculated by Equation 3 for the soil and roots samples

| **Sample** | **La** | **Ce** | **Pr** | **Nd** | **Sm** | **Eu** | **Gd** | **Dy** | **Tb** | **Ho** | **Er** | **Tm** | **Yb** | **Lu** | **Y** |
| --- | --- | --- | --- | --- | --- | --- | --- | --- | --- | --- | --- | --- | --- | --- | --- |
| PFC001 | 0.0298 | 0.0201 | 0.0209 | 0.0226 | 0.0168 | 0.0162 | 0.0186 | 0.0176 | 0.0151 | 0.0203 | 0.0195 | 0.0186 | 0.0179 | 0.0150 | 0.0205 |
| PFC002 | 0.0306 | 0.0242 | 0.0238 | 0.0249 | 0.0229 | 0.0193 | 0.0218 | 0.0244 | 0.0194 | 0.0231 | 0.0254 | 0.0240 | 0.0286 | 0.0260 | 0.0289 |
| PFC003 | 0.0833 | 0.0642 | 0.0615 | 0.0655 | 0.0532 | 0.0473 | 0.0479 | 0.0510 | 0.0429 | 0.0496 | 0.0520 | 0.0435 | 0.0536 | 0.0502 | 0.0578 |
| PFC004 | 0.0767 | 0.0641 | 0.0591 | 0.0615 | 0.0494 | 0.0432 | 0.0458 | 0.0442 | 0.0389 | 0.0459 | 0.0418 | 0.0377 | 0.0486 | 0.0406 | 0.0484 |
| PFC005 | 0.0420 | 0.0292 | 0.0285 | 0.0306 | 0.0258 | 0.0246 | 0.0274 | 0.0302 | 0.0241 | 0.0343 | 0.0322 | 0.0333 | 0.0481 | 0.0408 | 0.0405 |
| PFC006 | 0.0405 | 0.0277 | 0.0279 | 0.0291 | 0.0247 | 0.0223 | 0.0251 | 0.0270 | 0.0215 | 0.0267 | 0.0267 | 0.0263 | 0.0343 | 0.0324 | 0.0309 |
| PFC007 | 0.0153 | 0.0113 | 0.0115 | 0.0120 | 0.0103 | 0.0092 | 0.0095 | 0.0099 | 0.0086 | 0.0095 | 0.0093 | 0.0089 | 0.0096 | 0.0103 | 0.0110 |
| PFC008 | 0.0340 | 0.0271 | 0.0255 | 0.0274 | 0.0230 | 0.0211 | 0.0225 | 0.0233 | 0.0197 | 0.0233 | 0.0230 | 0.0255 | 0.0288 | 0.0242 | 0.0276 |
| PFC009 | 0.0707 | 0.0508 | 0.0468 | 0.0476 | 0.0386 | 0.0349 | 0.0379 | 0.0404 | 0.0331 | 0.0403 | 0.0413 | 0.0399 | 0.0492 | 0.0466 | 0.0478 |
| PFC010 | 0.0424 | 0.0286 | 0.0273 | 0.0278 | 0.0220 | 0.0198 | 0.0223 | 0.0220 | 0.0179 | 0.0213 | 0.0213 | 0.0201 | 0.0232 | 0.0253 | 0.0254 |
| PFC011 | 0.0273 | 0.0225 | 0.0243 | 0.0272 | 0.0290 | 0.0368 | 0.0367 | 0.0531 | 0.0366 | 0.0596 | 0.0642 | 0.0765 | 0.0638 | 0.0670 | 0.0650 |
| PFC012 | 0.0107 | 0.0084 | 0.0091 | 0.0093 | 0.0087 | 0.0100 | 0.0091 | 0.0123 | 0.0097 | 0.0138 | 0.0170 | 0.0130 | 0.0159 | 0.0180 | 0.0182 |
| PFC013 | 0.0314 | 0.0266 | 0.0269 | 0.0284 | 0.0300 | 0.0343 | 0.0341 | 0.0507 | 0.0401 | 0.0558 | 0.0576 | 0.0605 | 0.0642 | 0.0465 | 0.0648 |
| PFC014 | 0.0132 | 0.0101 | 0.0107 | 0.0116 | 0.0108 | 0.0161 | 0.0154 | 0.0251 | 0.0169 | 0.0254 | 0.0321 | 0.0257 | 0.0352 | 0.0310 | 0.0351 |
| PFC015 | 0.0424 | 0.0321 | 0.0334 | 0.0352 | 0.0361 | 0.0553 | 0.0385 | 0.0561 | 0.0383 | 0.0660 | 0.0771 | 0.0573 | 0.0767 | 0.0540 | 0.0744 |
| PTC001 | 0.0139 | 0.0126 | 0.0129 | 0.0148 | 0.0135 | 0.0125 | 0.0140 | 0.0156 | 0.0135 | 0.0160 | 0.0157 | 0.0154 | 0.0166 | 0.0154 | 0.0172 |
| PTC002 | 0.0123 | 0.0073 | 0.0096 | 0.0103 | 0.0082 | 0.0080 | 0.0091 | 0.0086 | 0.0070 | 0.0078 | 0.0073 | 0.0070 | 0.0083 | 0.0063 | 0.0099 |
| PTC003 | 0.0756 | 0.0612 | 0.0615 | 0.0673 | 0.0560 | 0.0532 | 0.0579 | 0.0531 | 0.0479 | 0.0508 | 0.0483 | 0.0453 | 0.0448 | 0.0373 | 0.0539 |
| PTC004 | 0.0118 | 0.0093 | 0.0100 | 0.0108 | 0.0093 | 0.0089 | 0.0098 | 0.0110 | 0.0090 | 0.0104 | 0.0104 | 0.0106 | 0.0117 | 0.0090 | 0.0118 |
| PTC005 | 0.0174 | 0.0130 | 0.0140 | 0.0153 | 0.0126 | 0.0118 | 0.0134 | 0.0147 | 0.0119 | 0.0145 | 0.0143 | 0.0140 | 0.0154 | 0.0133 | 0.0158 |
| PTC006 | 0.0199 | 0.0135 | 0.0127 | 0.0120 | 0.0078 | 0.0064 | 0.0072 | 0.0082 | 0.0060 | 0.0083 | 0.0080 | 0.0072 | 0.0090 | 0.0105 | 0.0088 |
| PTC007 | 0.0195 | 0.0164 | 0.0172 | 0.0194 | 0.0178 | 0.0164 | 0.0189 | 0.0210 | 0.0168 | 0.0200 | 0.0194 | 0.0188 | 0.0229 | 0.0188 | 0.0220 |
| PTC008 | 0.0252 | 0.0211 | 0.0211 | 0.0219 | 0.0200 | 0.0164 | 0.0183 | 0.0177 | 0.0159 | 0.0175 | 0.0161 | 0.0148 | 0.0158 | 0.0134 | 0.0188 |
| PTC009 | 0.0177 | 0.0132 | 0.0142 | 0.0150 | 0.0123 | 0.0120 | 0.0134 | 0.0128 | 0.0113 | 0.0121 | 0.0090 | 0.0093 | 0.0091 | 0.0075 | 0.0127 |
| PTC010 | 0.0186 | 0.0147 | 0.0164 | 0.0186 | 0.0171 | 0.0166 | 0.0187 | 0.0179 | 0.0161 | 0.0173 | 0.0158 | 0.0147 | 0.0162 | 0.0130 | 0.0203 |

**Table S6** TF, calculated by Equation 4 for the leaves and roots samples

| **Sample** | **La** | **Ce** | **Pr** | **Nd** | **Sm** | **Eu** | **Gd** | **Dy** | **Tb** | **Ho** | **Er** | **Tm** | **Yb** | **Lu** | **Y** |
| --- | --- | --- | --- | --- | --- | --- | --- | --- | --- | --- | --- | --- | --- | --- | --- |
| PFC001 | 0.242 | 0.264 | 0.246 | 0.250 | 0.292 | 0.240 | 0.260 | 0.280 | 0.270 | 0.246 | 0.255 | 0.237 | 0.291 | 0.200 | 0.0205 |
| PFC002 | 0.046 | 0.033 | 0.039 | 0.041 | 0.042 | 0.042 | 0.039 | 0.037 | 0.042 | 0.045 | 0.034 | 0.038 | 0.042 | 0.034 | 0.0289 |
| PFC003 | 0.027 | 0.009 | 0.019 | 0.019 | 0.017 | 0.018 | 0.021 | 0.017 | 0.018 | 0.022 | 0.023 | 0.020 | 0.019 | 0.000 | 0.0578 |
| PFC004 | 0.023 | 0.015 | 0.018 | 0.016 | 0.019 | 0.016 | 0.015 | 0.015 | 0.015 | 0.016 | 0.016 | 0.013 | 0.010 | 0.000 | 0.0484 |
| PFC005 | 0.042 | 0.036 | 0.037 | 0.038 | 0.037 | 0.034 | 0.040 | 0.040 | 0.039 | 0.039 | 0.034 | 0.040 | 0.036 | 0.031 | 0.0405 |
| PFC006 | 0.043 | 0.033 | 0.040 | 0.040 | 0.039 | 0.038 | 0.036 | 0.036 | 0.033 | 0.036 | 0.027 | 0.033 | 0.039 | 0.031 | 0.0309 |
| PFC007 | 0.074 | 0.045 | 0.062 | 0.060 | 0.057 | 0.050 | 0.055 | 0.061 | 0.050 | 0.071 | 0.072 | 0.065 | 0.091 | 0.000 | 0.0110 |
| PFC008 | 0.069 | 0.063 | 0.069 | 0.069 | 0.067 | 0.069 | 0.067 | 0.070 | 0.065 | 0.067 | 0.076 | 0.074 | 0.069 | 0.076 | 0.0276 |
| PFC009 | 0.115 | 0.124 | 0.119 | 0.120 | 0.117 | 0.113 | 0.115 | 0.127 | 0.117 | 0.124 | 0.123 | 0.144 | 0.129 | 0.133 | 0.0478 |
| PFC010 | 0.182 | 0.182 | 0.180 | 0.186 | 0.178 | 0.185 | 0.187 | 0.184 | 0.173 | 0.185 | 0.171 | 0.186 | 0.171 | 0.168 | 0.0254 |
| PFC011 | 0.212 | 0.154 | 0.177 | 0.162 | 0.880 | 0.128 | 0.117 | 0.088 | 0.094 | 0.115 | 0.090 | 0.092 | 0.052 | 0.067 | 0.0650 |
| PFC012 | 0.387 | 0.340 | 0.303 | 0.331 | 0.307 | 0.332 | 0.347 | 0.342 | 0.264 | 0.362 | 0.309 | 0.385 | 0.329 | 0.444 | 0.0182 |
| PFC013 | 0.129 | 0.067 | 0.099 | 0.094 | 0.075 | 0.095 | 0.108 | 0.110 | 0.105 | 0.113 | 0.119 | 0.140 | 0.084 | 0.118 | 0.0648 |
| PFC014 | 0.327 | 0.199 | 0.284 | 0.268 | 0.248 | 0.238 | 0.207 | 0.188 | 0.189 | 0.208 | 0.179 | 0.143 | 0.140 | 0.145 | 0.0351 |
| PFC015 | 0.090 | 0.060 | 0.069 | 0.068 | 0.053 | 0.050 | 0.049 | 0.048 | 0.047 | 0.041 | 0.043 | 0.041 | 0.034 | 0.043 | 0.0744 |
| PTC001 | 0.039 | 0.026 | 0.037 | 0.038 | 0.042 | 0.037 | 0.043 | 0.034 | 0.038 | 0.047 | 0.047 | 0.041 | 0.045 | 0.049 | 0.0608 |
| PTC002 | 0.315 | 0.299 | 0.354 | 0.341 | 0.441 | 0.258 | 0.292 | 0.312 | 0.330 | 0.333 | 0.418 | 0.314 | 0.228 | 0.280 | 0.0032 |
| PTC003 | 0.042 | 0.048 | 0.047 | 0.044 | 0.049 | 0.053 | 0.030 | 0.037 | 0.035 | 0.041 | 0.032 | 0.033 | 0.039 | 0.000 | 0.0174 |
| PTC004 | 0.071 | 0.050 | 0.054 | 0.051 | 0.057 | 0.036 | 0.048 | 0.041 | 0.038 | 0.050 | 0.049 | 0.041 | 0.036 | 0.185 | 0.2829 |
| PTC005 | 0.133 | 0.068 | 0.112 | 0.116 | 0.120 | 0.123 | 0.122 | 0.107 | 0.105 | 0.107 | 0.088 | 0.102 | 0.072 | 0.000 | 0.0106 |
| PTC006 | 0.068 | 0.072 | 0.094 | 0.099 | 0.319 | 0.125 | 0.102 | 0.120 | 0.119 | 0.128 | 0.110 | 0.111 | 0.146 | 0.214 | 0.0107 |
| PTC007 | 0.097 | 0.068 | 0.096 | 0.103 | 0.094 | 0.088 | 0.096 | 0.086 | 0.091 | 0.088 | 0.083 | 0.073 | 0.062 | 0.000 | 0.0476 |
| PTC008 | 0.079 | 0.077 | 0.070 | 0.070 | 0.134 | 0.058 | 0.058 | 0.065 | 0.054 | 0.048 | 0.035 | 0.027 | 0.043 | 0.000 | 0.0124 |
| PTC009 | 0.289 | 0.172 | 0.230 | 0.260 | 0.246 | 0.194 | 0.207 | 0.164 | 0.168 | 0.156 | 0.169 | 0.162 | 0.106 | 0.233 | 0.0054 |
| PTC010 | 0.318 | 0.134 | 0.345 | 0.354 | 0.296 | 0.288 | 0.306 | 0.229 | 0.245 | 0.247 | 0.224 | 0.214 | 0.173 | 0.000 | 0.0240 |

**Table S7** Concentration (mg kg^‒1^) of macronutrients and micronutrients correlated with REEs in *B. trimera*. The letters P and R identify the leaves and root samples, respectively

| **Samples** | **Ca** | **Fe** | **P** | **Mn** | **Mg** | **K** | **Al** | **Cu** | **ΣLREEs** | **ΣHREEs** |
| --- | --- | --- | --- | --- | --- | --- | --- | --- | --- | --- |
| PFC001P | 6320 | 524 | 1740 | 148 | 2040 | 15100 | 334 | 12.5 | 1.685 | 0.312 |
| PFC002P | 6590 | 183 | 2160 | 145 | 2740 | 16500 | 133 | 14.4 | 1.658 | 0.220 |
| PFC003P | 7950 | 127 | 2510 | 204 | 2760 | 17200 | 83 | 11.6 | 1.306 | 0.194 |
| PFC004P | 6310 | 130 | 2780 | 268 | 2370 | 16300 | 79 | 8.81 | 1.107 | 0.105 |
| PFC005P | 7020 | 248 | 2040 | 142 | 2290 | 16400 | 159 | 6.76 | 1.747 | 0.158 |
| PFC006P | 5450 | 209 | 1940 | 156 | 2070 | 12400 | 119 | 9.26 | 1.408 | 0.170 |
| PFC007P | 6010 | 152 | 3070 | 101 | 2260 | 16200 | 104 | 9.17 | 0.955 | 0.089 |
| PFC008P | 7060 | 347 | 2290 | 77.1 | 2440 | 18700 | 230 | 15.3 | 2.915 | 0.273 |
| PFC009P | 8710 | 1530 | 2380 | 160 | 3340 | 19600 | 788 | 16.4 | 8.255 | 0.744 |
| PFC010P | 7690 | 924 | 2410 | 107 | 3020 | 21200 | 681 | 14.1 | 5.665 | 0.657 |
| PFC011P | 5930 | 221 | 1090 | 504 | 2410 | 17500 | 200 | 14 | 1.631 | 0.270 |
| PFC012P | 7950 | 170 | 1170 | 571 | 2020 | 18500 | 147 | 11.2 | 1.040 | 0.156 |
| PFC013P | 4960 | 171 | 1160 | 223 | 2060 | 15200 | 168 | 9.52 | 0.871 | 0.290 |
| PFC014P | 7900 | 215 | 1510 | 636 | 2750 | 20000 | 187 | 10.7 | 0.989 | 0.230 |
| PFC015P | 6640 | 128 | 1470 | 355 | 2520 | 19400 | 109 | 9.49 | 0.642 | 0.136 |
| PFC016P | 8080 | 108 | 1540 | 321 | 2800 | 12600 | 96 | 20.4 | 0.999 | 0.162 |
| PFC017P | 6110 | 166 | 2520 | 256 | 2680 | 18300 | 143 | 18 | 1.255 | 0.165 |
| PFC018P | 6780 | 243 | 2420 | 202 | 2680 | 13000 | 78 | 86.2 | 0.804 | 0.102 |
| PFC019P | 5560 | 127 | 1770 | 358 | 2840 | 24200 | 95 | 21.5 | 1.145 | 0.267 |
| PTC001P | 6600 | 97 | 2770 | 100 | 2390 | 18700 | 52 | 12 | 0.673 | 0.149 |
| PTC002P | 7100 | 165 | 1930 | 108 | 2650 | 16500 | 109 | 11.5 | 1.316 | 0.204 |
| PTC003P | 6850 | 122 | 1390 | 39.4 | 3680 | 16400 | 82 | 12.2 | 0.641 | 0.097 |
| PTC004P | 6130 | 64 | 1920 | 99.9 | 2560 | 16000 | 35 | 10.6 | 0.498 | 0.062 |
| PTC005P | 5980 | 146 | 2100 | 84.7 | 2280 | 24700 | 76 | 14.9 | 1.306 | 0.236 |
| PTC006P | 7380 | 119 | 1800 | 42.6 | 2900 | 26000 | 89 | 9.02 | 0.666 | 0.077 |
| PTC007P | 6190 | 145 | 2950 | 113 | 2620 | 20400 | 93 | 12.4 | 0.099 | 0.236 |
| PTC008P | 6430 | 67 | 1860 | 193 | 2750 | 11600 | 49 | 10.8 | 0.025 | 0.065 |
| PTC009P | 6330 | 62 | 1680 | 115 | 2630 | 15900 | 47 | 10.2 | 0.043 | 0.119 |
| PTC010P | 8440 | 82 | 2980 | 164 | 2390 | 10100 | 60 | 15 | 0.202 | 0.539 |
| PFC001R | 5930 | 1460 | 1250 | 315 | 1900 | 13100 | 1040 | 21.8 | 6.618 | 1.193 |
| PFC002R | 6160 | 3590 | 1290 | 382 | 2460 | 11100 | 2460 | 27.6 | 42.961 | 4.730 |
| PFC003R | 6830 | 8660 | 2580 | 280 | 3110 | 15300 | 5320 | 66.7 | 79.696 | 6.644 |
| PFC004R | 5030 | 8790 | 1300 | 367 | 2270 | 6390 | 5840 | 27.8 | 65.055 | 6.563 |
| PFC005R | 5190 | 6130 | 1900 | 210 | 2760 | 15600 | 4200 | 57.1 | 45.699 | 3.916 |
| PFC006R | 6960 | 5110 | 1590 | 383 | 3610 | 15200 | 3090 | 52.7 | 37.302 | 3.903 |
| PFC007R | 2440 | 1600 | 1080 | 64.6 | 1120 | 14900 | 992 | 17.3 | 16.705 | 1.253 |
| PFC008R | 7870 | 3060 | 2260 | 184 | 2410 | 16200 | 2060 | 32.5 | 43.781 | 3.775 |
| PFC009R | 6900 | 10400 | 2490 | 274 | 3470 | 13000 | 5780 | 69.8 | 68.684 | 5.956 |
| PFC010R | 7290 | 4640 | 2100 | 198 | 2880 | 12600 | 3160 | 33.2 | 31.020 | 3.618 |
| PFC011R | 3290 | 4640 | 792 | 567 | 1890 | 16800 | 4060 | 33.5 | 8.104 | 2.056 |
| PFC012R | 3730 | 1020 | 595 | 295 | 1150 | 14000 | 972 | 14.7 | 3.000 | 0.373 |
| PFC013R | 3950 | 3920 | 759 | 296 | 2180 | 15900 | 3930 | 21.5 | 9.769 | 1.687 |
| PFC014R | 2820 | 1460 | 805 | 281 | 1550 | 14800 | 1540 | 19.3 | 3.945 | 0.976 |
| PFC015R | 4270 | 3060 | 735 | 643 | 2000 | 13400 | 3980 | 29.2 | 9.234 | 2.117 |
| PTC001R | 5050 | 1560 | 1510 | 165 | 1560 | 15100 | 1140 | 16.5 | 20.380 | 2.710 |
| PTC002R | 2990 | 655 | 986 | 74.8 | 1160 | 11800 | 328 | 13 | 4.099 | 0.579 |
| PTC003R | 6920 | 2230 | 1230 | 112 | 2200 | 9680 | 2010 | 26.3 | 14.123 | 2.554 |
| PTC004R | 3600 | 924 | 1130 | 120 | 1310 | 15600 | 601 | 19.3 | 8.828 | 1.128 |
| PTC005R | 2330 | 1530 | 996 | 76.7 | 1100 | 11100 | 986 | 21.3 | 13.012 | 1.626 |
| PTC006R | 4700 | 1340 | 1050 | 70.7 | 2500 | 15800 | 1050 | 12.8 | 8.225 | 0.645 |
| PTC007R | 3450 | 1930 | 1720 | 143 | 1720 | 12300 | 1340 | 21.7 | 19.677 | 2.400 |
| PTC008R | 5460 | 978 | 1200 | 428 | 2080 | 6770 | 824 | 20.2 | 5.970 | 1.114 |
| PTC009R | 2080 | 385 | 764 | 67.8 | 926 | 10900 | 371 | 15.3 | 4.023 | 0.548 |
| PTC010R | 3910 | 1350 | 1490 | 161 | 1480 | 5760 | 948 | 18.6 | 12.727 | 1.736 |

**Table S8** Concentration of REEs (mg kg^‒1^) in rock samples

|  | **Samples** | **La** | **Ce** | **Pr** | **Nd** | **Sm** | **Eu** | **Gd** | **Tb** | **Dy** | **Ho** | **Er** | **Tm** | **Yb** | **Lu** | **Y** | **LREEs** | **HREEs** | **Ce*** | **Eu*** |
| --- | --- | --- | --- | --- | --- | --- | --- | --- | --- | --- | --- | --- | --- | --- | --- | --- | --- | --- | --- | --- |
| PFC | PPF-20/04B | 335.0 | 676.0 | 84.0 | 319.0 | 51.3 | 12.6 | 34.8 | 4.3 | 19.4 | 3.1 | 6.9 | 0.9 | 4.6 | 0.6 | 82.0 | 1477.9 | 156.5 | 0.928 | 1.293 |
|  | PPF-21/14C | 279.0 | 537.0 | 65.0 | 242.0 | 37.5 | 9.5 | 26.5 | 3.4 | 16.1 | 2.5 | 6.7 | 0.8 | 5.5 | 0.7 | 76.3 | 1170.0 | 138.5 | 0.913 | 1.300 |
|  | PPF-21/14B | 208.0 | 389.0 | 44.3 | 168.0 | 24.7 | 6.9 | 18.4 | 2.4 | 11.2 | 1.9 | 4.7 | 0.6 | 3.5 | 0.5 | 52.4 | 840.9 | 95.4 | 0.908 | 1.401 |
|  | PPF-21/14 | 238.0 | 450.0 | 53.9 | 196.5 | 29.3 | 8.0 | 21.4 | 2.8 | 13.0 | 2.1 | 5.4 | 0.7 | 4.1 | 0.5 | 61.4 | 975.7 | 111.4 | 0.911 | 1.382 |
|  | PCB-5A1 | 282.0 | 596.0 | 67.4 | 257.0 | 35.6 | 10.1 | 28.2 | 3.5 | 15.2 | 2.6 | 5.6 | 0.6 | 3.9 | 0.6 | 65.1 | 1248.1 | 125.2 | 0.987 | 1.382 |
|  | PCB-47A(L) | 256.0 | 519.0 | 57.8 | 220.0 | 32.3 | 9.1 | 21.6 | 2.9 | 14.4 | 2.0 | 5.4 | 0.8 | 3.6 | 0.6 | 58.6 | 1094.2 | 109.9 | 0.965 | 1.497 |
|  | PCB-47A(G) | 281.0 | 592.0 | 67.2 | 255.0 | 38.9 | 11.2 | 27.2 | 3.4 | 15.8 | 2.6 | 6.0 | 0.7 | 3.7 | 0.6 | 66.9 | 1245.3 | 126.8 | 0.985 | 1.493 |
|  | PCB-47A | 281.0 | 629.0 | 70.1 | 269.0 | 44.5 | 11.6 | 30.7 | 3.5 | 15.9 | 2.6 | 5.6 | 0.7 | 4.0 | 0.5 | 71.3 | 1305.2 | 134.7 | 1.027 | 1.355 |
|  | PPF-20/04A | 312.0 | 696.0 | 78.7 | 313.0 | 44.7 | 11.7 | 29.5 | 3.4 | 17.7 | 2.5 | 5.6 | 0.7 | 3.6 | 0.5 | 67.5 | 1456.1 | 131.1 | 1.007 | 1.391 |
|  | PPF-20/14A | 288.0 | 600.0 | 68.3 | 245.0 | 42.7 | 10.7 | 27.4 | 3.3 | 15.8 | 2.5 | 5.6 | 0.7 | 4.0 | 0.6 | 66.6 | 1254.7 | 126.4 | 0.995 | 1.356 |
|  | **Minimum** | 208.0 | 389.0 | 44.3 | 168.0 | 24.7 | 6.9 | 18.4 | 2.4 | 11.2 | 1.9 | 4.7 | 0.6 | 3.5 | 0.5 | 52.4 | 840.9 | 95.4 | 0.908 | 1.293 |
|  | **Mean** | 276.0 | 568.4 | 65.7 | 248.5 | 38.2 | 10.1 | 26.6 | 3.3 | 15.4 | 2.4 | 5.7 | 0.7 | 4.0 | 0.6 | 66.8 | 1206.8 | 125.6 | 0.963 | 1.385 |
|  | **Maximum** | 335.0 | 696.0 | 84.0 | 319.0 | 51.3 | 12.6 | 34.8 | 4.3 | 19.4 | 3.1 | 6.9 | 0.9 | 5.5 | 0.7 | 82.0 | 1477.9 | 156.5 | 1.027 | 1.497 |
| PTC | PTC-1A | 270.0 | 634.0 | 72.5 | 296.0 | 47.4 | 13.2 | 35.8 | 4.7 | 22.5 | 3.6 | 8.4 | 1.1 | 6.3 | 0.9 | 84.7 | 1333.1 | 167.98 | 1.026 | 1.389 |
|  | PTC-1B | 280.0 | 609.0 | 71.7 | 274.0 | 48.9 | 13.0 | 35.2 | 4.6 | 23.3 | 3.9 | 9.7 | 1.2 | 7.2 | 1.0 | 89.2 | 1296.6 | 175.226 | 0.991 | 1.359 |
|  | PT-T-G | 311.0 | 702.0 | 82.5 | 318.0 | 54.7 | 15.6 | 39.1 | 4.9 | 25.0 | 4.1 | 9.8 | 1.2 | 7.0 | 0.9 | 101.0 | 1483.8 | 192.941 | 1.012 | 1.463 |
|  | PT-T-F | 321.0 | 662.0 | 76.0 | 294.0 | 47.0 | 13.0 | 33.9 | 4.3 | 21.5 | 3.7 | 9.0 | 1.1 | 6.4 | 0.9 | 91.8 | 1413 | 172.557 | 0.961 | 1.412 |
|  | PT-T-A | 305.0 | 667.0 | 79.8 | 308.0 | 53.4 | 15.1 | 38.0 | 4.8 | 24.2 | 4.0 | 9.7 | 1.2 | 6.9 | 0.9 | 101.0 | 1428.3 | 190.705 | 0.985 | 1.453 |
|  | PT-T-B | 332.0 | 746.0 | 86.0 | 322.0 | 54.7 | 15.7 | 38.3 | 4.8 | 24.1 | 3.9 | 9.6 | 1.2 | 7.0 | 0.9 | 97.9 | 1556.4 | 187.649 | 1.027 | 1.487 |
|  | PT-T-C | 284.0 | 671.0 | 75.6 | 311.0 | 52.0 | 14.1 | 36.9 | 4.5 | 21.8 | 3.6 | 8.2 | 1.0 | 5.5 | 0.8 | 92.7 | 1407.7 | 174.842 | 1.033 | 1.396 |
|  | PT-M-A | 260.0 | 680.0 | 80.1 | 307.0 | 53.8 | 16.4 | 36.9 | 4.7 | 22.2 | 3.5 | 8.3 | 1.1 | 5.8 | 0.8 | 90.7 | 1397.3 | 173.874 | 1.110 | 1.596 |
|  | PT-M-B | 291.0 | 688.0 | 80.7 | 315.0 | 53.7 | 15.1 | 37.2 | 4.7 | 22.1 | 3.6 | 8.5 | 1.1 | 6.2 | 0.8 | 89.8 | 1443.5 | 174.007 | 1.038 | 1.465 |
|  | **Minimum** | 260 | 609 | 71.7 | 274 | 47 | 13 | 33.9 | 4.2 | 21.5 | 3.5 | 8.1 | 1.0 | 5.4 | 0.75 | 84.7 | 1296.6 | 167.98 | 0.961 | 1.359 |
|  | **Mean** | 294.89 | 673.2 | 78.3 | 305.0 | 51.7 | 14.5 | 36.8 | 4.6 | 22.9 | 3.7 | 9.0 | 1.1 | 6.4 | 0.87 | 93.2 | 1417.7 | 178.864 | 1.020 | 1.447 |
|  | **Maximum** | 332 | 746 | 86 | 322 | 54.7 | 16.4 | 39.1 | 4.9 | 25 | 4.0 | 9.7 | 1.1 | 7.1 | 0.98 | 101 | 1556.4 | 192.941 | 1.110 | 1.596 |

**Table S9** Pearson correlation values between environmental matrices

| **Rock-**  **soil** | **Soil-**  **roots** | **Roots- leave** | **Carbonatite- roots** | **Carbonatite- leaves** | **Soil-leaves** |
| --- | --- | --- | --- | --- | --- |
| 0.998 | 0.994 | 0.962 | 0.991 | 0.923 | 0.925 |

**References**

Alfaro MR, Nascimento CWA, Biondi CM, Silva YJAB, Silva YJAB, Accioly AMA, Montero A, Ugarte OM, Estevez J (2018) Rare-earth-element geochemistry in soils developed in different geological settings of Cuba. CATENA 162:317–324. https://doi.org/10.1016/j.catena.2017.10.031

Bamforth TG, Xia F, Putnis A, Brugger J, Hu S-Y, Roberts MP, Suvorova A, Pring A (2024) Hydrothermal mineral replacement in the apatite-rhabdophane-monazite system: Experiments, reaction mechanisms and geological implications. Chem Geol 666:122307. https://doi.org/10.1016/j.chemgeo.2024.122307

Brioschi L, Steinmann M, Lucot E, Pierret MC, Stille P, Prunier J, Badot PM (2013) Transfer of rare earth elements (REE) from natural soil to plant systems: Implications for the environmental availability of anthropogenic REE. Plant Soil 366:143–163. https://doi.org/10.1007/s11104-012-1407-0

Cerva-Alves T, Remus MVD, Dani N, Basei MAS (2017) Integrated field, mineralogical and geochemical characteristics of Caçapava do Sul alvikite and beforsite intrusions: A new Ediacaran carbonatite complex in southernmost Brazil. Ore Geol Rev 88:352–369. https://doi.org/10.1016/j.oregeorev.2017.05.017

Clavier N, Mesbah A, Szenknect S, Dacheux N (2018) Monazite, rhabdophane, xenotime & churchite: Vibrational spectroscopy of gadolinium phosphate polymorphs. Spectrochim Acta A Mol Biomol Spectrosc 205:85–94. https://doi.org/10.1016/j.saa.2018.07.016

Guastoni A, Secco L, Škoda R, Nestola F, Schiazza M, Novák M, Pennacchioni G (2019) Non-Metamict Aeschynite-(Y), Polycrase-(Y), and Samarskite-(Y) in NYF Pegmatites from Arvogno, Vigezzo Valley (Central Alps, Italy). Minerals 9:1–23. https://doi.org/10.3390/min9050313

Landim JSP, Silva YJAB, Nascimento CWA, Silva YJAB, Nascimento RC, Boechat CL, Silva CMCAC, Olinda RA, Barbosa RS, Silva TS, Biondi CM, Collins AL (2022) Distribution of rare earth elements in soils of contrasting geological and pedological settings to support human health assessment and environmental policies. Environ Geochem Health 44:861–872. https://doi.org/10.1007/s10653-021-00993-0

Morales BAA, Almeida DDPM, Koester E, Dorneles NT, Rosa MB, Martins AA (2019) Mineralogy, whole-rock geochemistry and C, O isotopes from Passo Feio Carbonatite, Sul-Riograndense Shield, Brazil. J South Am Earth Sci 94:102208. https://doi.org/10.1016/j.jsames.2019.05.024

Nasir S, Theye T, Massonne H-J (2009) REE-Rich Aeschynite in Apatite-Dolomite Carbonatite, Eastern Oman Mountains. Open Mineral J 3:17–27.

Penel G, Leroy G, Rey C, Sombret B, Huvenne JP, Bres E (1997) Infrared and Raman microspectrometry study of fluor-fluor-hydroxy and hydroxy-apatite powders. J Mater Sci Mater Med 8:271–276. https://doi.org/10.1023/A:1018504126866

Saminpanya S, Denkitkul N (2020) Micromorphology, mineralogy, and geochemistry of sediments at the tham lod rock shelter archaeological site in Mae Hong Son, Thailand: Suggestions of a late pleistocene climate. J Cave Karst Stud 82:51–68. DOI:10.4311/2019ES0111

Wei FS, Zheng CJ, Chen JS, Wu YY (1991) Study on the background contents on 61 elements of soils in China. Chin J Environ Sci 12:12–20
